# Deriving Vocal Fold Oscillation Information from Recorded Voice Signals Using Models of Phonation

**DOI:** 10.3390/e25071039

**Published:** 2023-07-10

**Authors:** Wayne Zhao, Rita Singh

**Affiliations:** 1 Department of Electrical and Computer Engineering, Carnegie Mellon University, Pittsburgh, PA 15213, USA; wenbozhao@cmu.edu; 2 School of Computer Science, Carnegie Mellon University, Pittsburgh, PA 15213, USA

**Keywords:** vocal fold oscillation, phonation models, dynamical systems, parameter estimation, voice profiling

## Abstract

During phonation, the vocal folds exhibit a self-sustained oscillatory motion, which is influenced by the physical properties of the speaker’s vocal folds and driven by the balance of bio-mechanical and aerodynamic forces across the glottis. Subtle changes in the speaker’s physical state can affect voice production and alter these oscillatory patterns. Measuring these can be valuable in developing computational tools that analyze voice to infer the speaker’s state. Traditionally, vocal fold oscillations (VFOs) are measured directly using physical devices in clinical settings. In this paper, we propose a novel analysis-by-synthesis approach that allows us to infer the VFOs directly from recorded speech signals on an individualized, speaker-by-speaker basis. The approach, called the ADLES-VFT algorithm, is proposed in the context of a joint model that combines a phonation model (with a glottal flow waveform as the output) and a vocal tract acoustic wave propagation model such that the output of the joint model is an estimated waveform. The ADLES-VFT algorithm is a forward-backward algorithm which minimizes the error between the recorded waveform and the output of this joint model to estimate its parameters. Once estimated, these parameter values are used in conjunction with a phonation model to obtain its solutions. Since the parameters correlate with the physical properties of the vocal folds of the speaker, model solutions obtained using them represent the individualized VFOs for each speaker. The approach is flexible and can be applied to various phonation models. In addition to presenting the methodology, we show how the VFOs can be quantified from a dynamical systems perspective for classification purposes. Mathematical derivations are provided in an appendix for better readability.

## 1. Introduction

Phonation is a complex bio-mechanical process wherein the glottal airflow, mediated by the muscles in the larynx and driven by an intricate balance of aerodynamic and mechanical forces across the glottis, maintains the vocal folds in a state of self-sustained vibration [1,2]. During this process, depending on the physical state of the vocal folds, their eigenmodes of vibration synchronize, or strive to do so. This self-sustained motion of the vocal folds during phonation is highly sensitive to perturbations caused by many possible influencing factors, which may affect the speaker during speech production. In recent years, there has been a surge of interest in building voice-based diagnostic aids—computational models based on artificial intelligence and machine learning that can infer the speaker’s state (and thereby the factors that are affecting the speaker) from voice. Such applications can benefit greatly from being able to deduce the fine-level nuances in the motion of the vocal folds, and being able to measure the response to various perturbing factors to infer their nature. However, doing this using traditional methods on an individual basis for each speaker is very difficult. Traditional methods to observe and record vocal fold oscillations (VFOs) are based on actual physical measurements taken using various instruments in clinical settings, e.g., [3,4,5].

The primary focus of this paper is to derive the VFOs for a speaker directly from recorded voice signals, alleviating the need for taking physical measurements. The solution we propose is an analysis-by-synthesis approach, based on physical models of phonation. We propose a methodology to deduce the parameters of a chosen phonation model from a speaker’s voice recording, which can then be substituted into a VFO model to obtain speaker-specific solutions, which represent the vocal fold oscillations of the speaker.

In the paragraphs below, we first review some relevant facts about the process of phonation. In the sections that follow, we present our proposed methodology in two stages: in the first, we show how we can infer the parameters of a physics-based model of phonation from measurements of glottal excitation. Since the parameters of such models represent the physical properties of the speaker’s vocal folds, using them to obtain solutions to the models gives us an estimate of the speaker’s VFOs. We subsequently show how to extend the model to include the physics of mucosal wave propagation in the vocal tract, and propose a forward-backward algorithm to estimate the parameters of the joint model. These can then be used in the corresponding phonation model to obtain its solutions.

Later on, we also explain how the solutions of these models (which are the deduced VFOs) can be characterized from a dynamical systems perspective to derive discriminative information in the form of features that are useful for classification tasks.

In this context, it is important to note at the outset that the models we use to demonstrate our methodology are simple and well-established models in the literature. It is *not* the goal of this paper to propose new models of phonation, but rather to propose a novel methodology to derive their parameters from speech signals, so that they can be solved to yield VFOs on a speaker-by-speaker basis. The main contribution of this paper lies in the derivation of the parameters of these models, and their individualized VFO solutions. The viability of these solutions is demonstrated experimentally using classification experiments.

### 1.1. The Bio-Mechanical Process of Phonation

**Figure 1:**
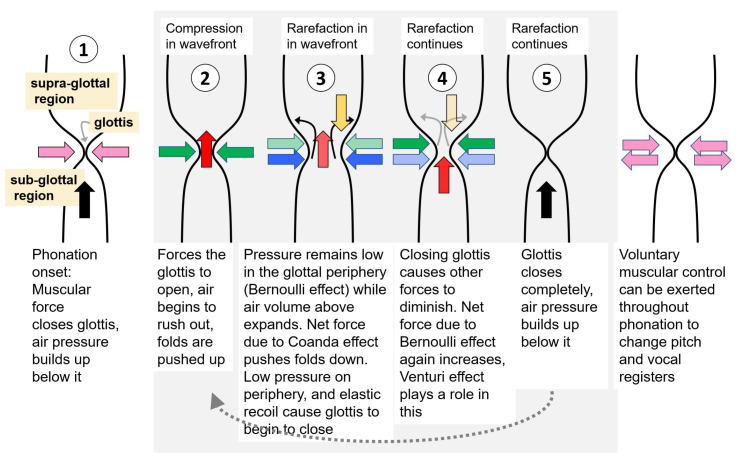
Schematic of the balance of some key forces through one cycle of the self-sustained vibrations of the vocal folds. Sequential “snapshots” of the cycle are numbered 1–5. Arrows depict the direction and type of forces in the glottal area. The color codes for the arrows depict net forces due to the following: Pink–muscular; Green–Bernoulli effect; Yellow–Coandǎ effect; Blue–vocal fold elasticity and other factors; Black and Red–air pressure. Lighter shades of each color depict weaker forces. Figure from [9] with permission.

By the myoelastic-aerodynamic theory of phonation, the forces in the laryngeal region that initiate and maintain phonation relate to (a) pressure balances and airflow dynamics within the supra-glottal and sub-glottal regions and (b) muscular control within the glottis and the larynx. The balance of forces necessary to cause self-sustained vibrations during phonation is created by physical phenomena such as the Bernoulli effect and the Coandǎ effect [6,7,8]. Figure 1 illustrates the interaction between these effects that is largely thought to drive the oscillations of the vocal folds.

The process of phonation begins with the closing of the glottis. This closure is voluntary and facilitated by the laryngeal muscles. Once closed, the muscles do not actively play a role in sustaining the vibrations. Glottal closure is followed by a contraction of the lungs which pushes out air and causes an increase in pressure just below the glottis. When this subglottal pressure crosses a threshold, the vocal folds are pushed apart, and air rushes out of the narrow glottal opening into the much wider supra-glottal region, creating negative intra-glottal pressure (with reference to atmospheric air pressure) [9].

The exact physics of the airflow through the glottis during phonation is well studied, e.g., [2,10,11,12,13,14]. The current understanding from these, from the airflow perspective, is that the glottis forms a flow separation plane. The air expansion in this region and the low pressure created in the vicinity of the glottis through the Coandǎ effect-induced entrainment cause a lowering of pressure close to the glottis and a net downward force on the glottis. At the same time, lowered pressure in the glottal region due to the Bernoulli effect that ensues from the high-velocity air volume flow through the glottis exerts a negative force on the glottis. The negative Bernoulli pressure causes elastic recoil, causing it to begin to close again. The closing reduces the volume flow through the glottis, diminishing the downward forces acting on it. Increased pressure build-up in the sub-glottal region causes the glottis to open again. This chain of oscillations continues in a self-sustained fashion throughout phonation until voluntary muscle control intervenes to alter or stop it or as the respiratory volume of air in the lungs is exhausted.

### 1.2. General Approaches to Phonation Modeling

Physical models of phonation, e.g., [9,11,15,16,17,18,19,20], attempt to explain this complex physical process using relations derived from actual physics, especially aerodynamics and the physics of mechanical structures.

For modeling purposes, we note that phonation is not the only source of excitation of the the vocal tract in producing speechsounds, which comprise both voiced and unvoiced sounds. However, phonation is indeed the primary source of excitation of the vocal tract in the production of *voiced* sounds, wherein the oscillation of the vocal folds modulates the pressure of the airflow to produce a (quasi-) periodic glottal flow wave at a fundamental frequency (the pitch), which in turn results in the occurrence of higher order harmonics. The resultant glottal flow further excites the vocal tract, which comprises the laryngeal cavity, the pharynx, and the oral and nasal cavities, to produce individual sounds. The vocal tract serves as a resonance chamber that produces formants. The identities of the different sounds produced within it are derived from these resonances, which in turn are largely dependent on the configurations of the vocal tract specified by their time-varying cross-sectional area.

From this perspective, phonation modeling has typically involved the modeling of two sub-processes: the self-sustained vibration of the vocal folds, and the propagation of the resultant pressure wave through the vocal tract [21]. Each sub-process model has associated parameters that determine the model output, given an input.

Following the above division of the process, we identify the two following model types, each modeling one of the sub-processes: (i) *Vocal fold oscillation (VFO) models* (also called *vocal folds models*, or *oscillation models*), and (ii) *vocal tract (VT) models*.

The VFO models describe the vibration of vocal folds and their aerodynamic interaction with airflow. Such models are of four broad types: one-mass models, e.g., [2,16,22,23,24], two-mass models, e.g., [11,15], multi-mass models [18], and finite element models, e.g., [17]. Each of these has proven to be useful in different contexts. The **VT** models describe the interaction of the glottal pressure wave with the vocal tract, which is turn has been described in the literature by varied models, such as statistical models, e.g., [25], geometric models, e.g., [26] and biomechanical models, e.g., [27].

In addition, different models are also applied to describe the aero-acoustic interaction of the glottal airflow and the vocal tract. Some examples of these are reflection-type line analog models and transmission line circuit analog models, e.g., [28], hybrid time-frequency domain models, e.g., [29] and finite-element models, e.g., [30].

## 2. The Problem of Parameter Estimation

Each of the models mentioned in Section 1.2 includes a set of parameters that determine its *state*, and *output*, given an input. For instance, given the parameters for a VFO model, the glottal flow waveform can be determined; given the glottal flow waveform as an input, and the parameters for a VFO model, the acoustic signal can be determined.

The problem we tackle in this paper is the *inverse* problem: given a model output, we must estimate the model parameters from it.

This can be of great practical use. For example, with speaker-specific parameter setting, the output of these models can be used as a proxy for the actual vocal fold motion of the corresponding speaker. To obtain the parameters of such models, the traditional solution has been to take actual physical measurements of the vocal fold oscillations, or of the glottal flow using techniques such as high-speed videostroboscopy, as mentioned in Section 1. This is not always feasible.

On the other hand, the inverse problem of parameter estimation of phonation models is quite difficult to solve through purely computational means. For example, in order to estimate the parameters of a vocal tract (VT) model, one must take into account the vocal tract coupling, the effect of the lossy medium that comprises the walls of the vocal tract, lip radiation, etc. Without serious approximations, the inverse problem in this case becomes eventually intractable. Some approaches simplify the solution by discretizing the vocal tract as a sequence of consecutive tubes of varying cross-sectional area, or with a mesh-grid. However, these approximations invariably increase the estimation error.

This paper proposes a methodology for solving the inverse problem of phonation models through purely computational means. As mentioned earlier, the methodology follows an analysis-by-synthesis approach. We explain this by first reviewing our previously proposed Adjoint Least Squares Estimation (ADLES) algorithm [31] that estimates the parameters of a VFO model by minimizing the error between a reference glottal flow waveform and the signal generated by the physical VFO model. We then describe our proposed ADLES-VFT algorithm to estimate the parameters of a *joint* VFO and VT model (also called a body-cover model). Instead of comparing the model-generated excitation at the glottis to a reference glottal flow, the flow is propagated through the vocal tract to generate a signal at the lips, which is compared to a recorded voice signal which is used as a reference. The algorithm proposed iteratively re-estimates the model parameters by minimizing the error between the reference voice sample and this generated signal.

Once estimated, these parameters are used with the VFO model to generate the speaker’s VFOs.

## 3. Vocal Folds, Vocal Tract and Joint Models

In this section we describe the VFO and VT models that we use as examples in this paper. We also explain the formulation of the joint model that we ultimately use to solve the inverse problem of parameter estimation.

### 3.1. The VFO Model

**Figure 2:**
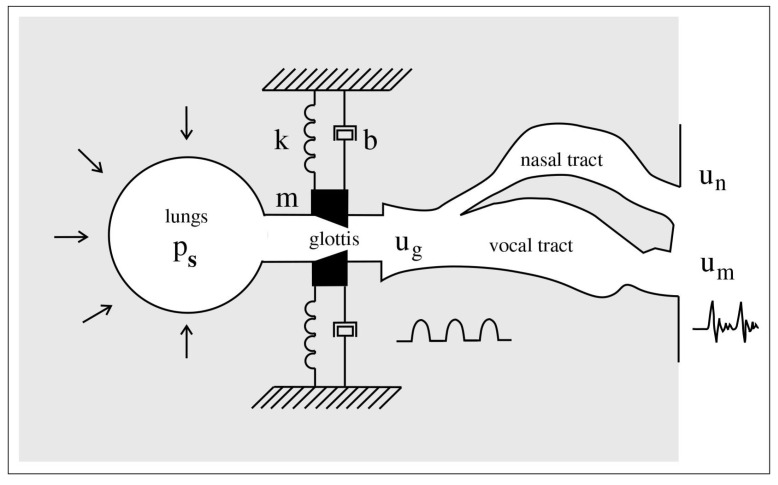
Approximating the vocal folds with mass-spring oscillators in the phonation process. Airflow from the lungs, driven by the subglottal pressure $P_{s}$, passes through the glottis, and vocal folds are set into a state of self-sustained vibration, producing the glottal flow $u_{g}$ which is a quasi-periodic pressure wave. The vibration of vocal folds is analogous to a pair of mass-spring-damper oscillators. Further, the glottal flow resonates in the speaker’s vocal tract and nasal tract and produces voiced sound.

A schematic illustration of a general mass-spring oscillator model for the vocal folds is shown in Figure 2. This is used to model the phonation process as described below.

One-mass models describe the vibration of the vocal folds as that of a single mass-damper-spring oscillator.

(1) $$M\ddot{x}+B\dot{x}+Kx=f\left(x,\dot{x},t\right)$$

where *x* is lateral displacement of a mass *M*, *B* and *K* are damping and stiffness coefficients, respectively, *f* is the driving force, and *t* is time [2]. The driving force is velocity-dependent and can be estimated by Bernoulli’s energy law:

(2) $$P_{g}=P_{s}-\frac{1}{2}\rho v^{2}$$

where $P_{g}$ is the mean glottal pressure, $P_{s}$ is sub-glottal pressure, ρ is air density, and *v* is the air particle velocity. The kinetic pressure in the supra-glottal region is neglected [2].

Other models, namely two-mass, multi-mass and finite element models can also be used as the basis for the VFO model, and are described briefly in the Appendix A for reference.

**Figure 3:**
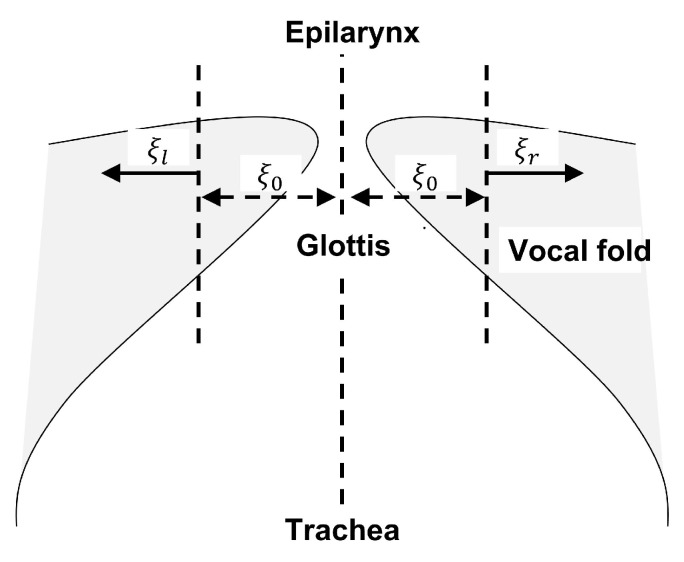
Diagram of the one-mass body-cover model for vocal folds. The lateral displacements at the midpoint of the left and right vocal folds are denoted as $\xi_{l}$ and $\xi_{r}$, and $\xi_{0}$ represents the half glottal width at rest.

For our paper, we adopt the version of the one-mass model of the vocal folds proposed in [24], illustrated in Figure 3. This is an asymmetric body-cover model which models the left and right vocal folds individually as one-mass components of a coupled dynamical system. It incorporates an asymmetry parameter, which can emulate the asymmetry in the vibratory motions of left and right vocal folds, and hence is also ideally suited to modeling pathological or atypical phonation [32].

The key assumptions made in formulating this model are:

(a) The degree of asymmetry is independent of the oscillation frequency;
(b) The glottal flow is stationary, frictionless, and incompressible;
(c) All subglottal and supraglottal loads are neglected, eliminating the effect of source-vocal tract interaction;
(d) There is no glottal closure and hence no vocal fold collision during the oscillation cycle;
(e) The small-amplitude body-cover assumption is applicable (see definition below).

**Assumption** **1** (Body-cover assumption). *The body-cover assumption assumes that a glottal flow-induced mucosal wave travels upwards within the transglottal region, causing a small displacement of the mucosal tissue, which attenuates down within a few millimeters into the tissue as an energy exchange happens between the airstream and the tissue [2].*

This assumption allows us to represent the mucosal wave as a one-dimensional surface wave on the mucosal surface (the cover) and treat the remainder of the vocal folds (the body) as a single mass or safely neglect it. As a result, the oscillation model can be linearized, and the oscillatory conditions are much simplified while maintaining the model’s accuracy.

In the one-mass asymmetric model proposed in [24], with reference to Figure 3, the center-line of the glottis is denoted as the *z*-axis. At the midpoint (z=0) of the thickness of the vocal folds, the left and right vocal folds oscillate with lateral displacement $\xi_{l}$ and $\xi_{r}$, resulting in a pair of coupled Van der Pol oscillators:

(3) $$\begin{array}{cc} \; & {\ddot{\xi }}_{r}+\beta \left(1+\xi_{r}^{2}\right){\dot{\xi }}_{r}+\xi_{r}-\frac{\Delta }{2}\xi_{r}=\alpha \left({\dot{\xi }}_{r}+{\dot{\xi }}_{l}\right)\\ \; & {\ddot{\xi }}_{l}+\beta \left(1+\xi_{l}^{2}\right){\dot{\xi }}_{l}+\xi_{l}+\frac{\Delta }{2}\xi_{l}=\alpha \left({\dot{\xi }}_{r}+{\dot{\xi }}_{l}\right) \end{array}$$

where β is the coefficient incorporating mass, spring and damping coefficients, α is the glottal pressure coupling coefficient, and Δ is the asymmetry coefficient. For a male adult with normal voice, the reference values for the model parameters (from clinical measurements) are usually approximately set to α=0.5, β=0.32 and Δ=0.

### 3.2. The VT Model

The literature describes a number of different approaches to modeling the vocal-tract, including bio-mechanical models, statistical models, and geometric models. For reference, they are described briefly in the Appendix B.

In our work we use an acoustic wave propagation model described by PDEs for the vocal tract. The vocal tract itself is represented as tube of length *L*, beginning at the glottis and ending at the lips. Representing the distance along the central axis of the vocal tract as *x*, x∈(0,L) (where x=0 at the glottis and x=L at the lips) and the time-varying volume velocity of air at any position *x* along the vocal tract as u(x,t), it can be shown that the PDE that describes u(x,t) is given by

(4) $$\frac{\partial^{2}u}{\partial t^{2}}=c^{2}\frac{\partial^{2}u}{\partial x^{2}}+f\left(x,t\right)$$

where f(x,t) represents the *vocal tract profile*, which models the characteristics of the vocal tract, including the effect of the nonuniform yielding wall on the acoustic flow dynamics, the effect of vocal tract coupling, lip radiation, etc., and must also be estimated by our algorithm. The derivation of Equation (4) is given in Appendix B. Note that if the vocal tract is assumed to be a rigid tube f(x,t)=0 and Equation (4) reduces to the well-known Webster-Horn equation [33]. In deriving Equation (4) we have assumed a static vocal tract, i.e., that the cross-sectional area A(x) of the vocal tract at any position *x* is constant. This assumption is valid during phonation, in particular during the steady state of sustained phonation; however our solution can also be extended to consider time-varying vocal tracts A(x,t), although we have not done so in this paper.

The oscillation of the vocal folds results in the movement of air with a time-varying volume velocity $u_{0}\left(t\right)=u\left(0,t\right)$ at the glottis. The vocal tract modulates this to result in the volume velocity $u_{L}\left(t\right)=u\left(L,t\right)$ at the lips and the corresponding pressure wave $p_{L}\left(t\right)$, where [34]

(5) $$u_{L}\left(t\right)=\frac{A(L)}{\rho c}p_{L}\left(t\right)$$

where A(L) is the opening area at the lip, *c* is the speed of sound, and ρ is the ambient air density.

## 4. Estimation of Model Parameters: Solving the Inverse Problem

Our objective is to derive vocal fold oscillations during phonation directly from the speech signal. In order to do so, we will utilize the VFO and VT models.

The *VFO* model represents the actual dynamics of the vocal folds. Given the model parameters, which are α, β and Δ for the coupled one-mass model of Equation (3), it can be used to compute the vocal fold oscillations and the volume velocity of air at the glottis. The *VT* model represents the dynamics of the vocal tract. Given an excitation (at the glottis), it can be used to compute the pressure wave at the lips, which manifests as sound.

Ours is the *inverse* problem: given the the pressure wave at the output of the vocal tract (i.e., the recorded speech signal) we must estimate the VFO parameters that could generate it. This is an *analysis-by-synthesis* problem: in order to analyze the given voice signal, we must identify the model parameters that synthesize the closest (in a metric sense) facsimile to it.

We present the solution in a two-step manner. In the first, given the actual excitation of the vocal tract to produce the voice signal, the parameters of the VFO model are estimated to minimize the error between its output and the reference excitation signal. We refer to this as the *backward* approach (and the corresponding estimation as the “backward” problem), since the reference excitation signal itself must first be derived by passing the voice signal through an inverse model of the vocal tract, i.e., “backward” through the vocal tract. We have previously described our solution to the backward problem in [31], and restate it in Section 4.1 for completeness.

The second, more complete solution considers the joint model, i.e., both the motions of the vocal folds and the propagation of the resulting air flow (the excitation) through the vocal tract. The model parameters are estimated by comparing the signal produced at the lips by the joint model to the recorded voice signal. We refer to this as the “forward-backward” approach since this requires forward propagating the output of the VFO through the VT model, prior to applying the backward approach. The solution to this problem is the primary contribution of this paper.

**Figure 4:**
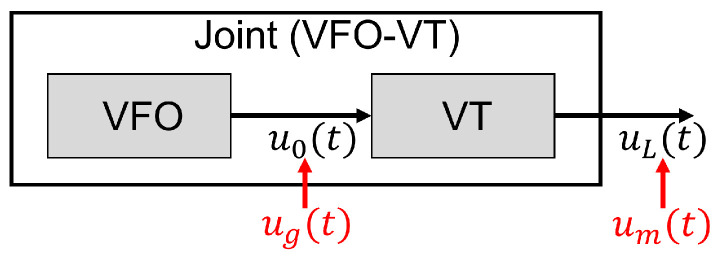
The VFO model models the generation of glottal signals by the movements of the vocal folds. The VT model models the transformation of the glottal signal generated by the vocal folds to the final voice signal. The joint VFO-VT model combines the two, using the output of the VFO model as the input to the VT model. ADLES compares the glottal signal $u_{0}\left(t\right)$ generated by the VFO model to a reference glottal signal $u_{g}\left(t\right)$ to estimate VFO parameters. ADLES-VFT compares the output of the joint model, $u_{L}\left(t\right)$, to a reference signal $u_{m}\left(t\right)$ obtained from an actual voice recording, to estimate both VFO and VT parameters. The output of the VFO model is the desired vocal-fold oscillation.

The two approaches are illustrated in Figure 4.

Once estimated, the VFO model parameters can be used to compute the oscillations of the vocal folds and their phase-space trajectories.

### 4.1. Estimating VFO Parameters from the Excitation Signal: The Backward Approach (ADLES)

As the first step, we describe the backward problem: how to derive the VFO model parameters that best explain the glottal excitation for a given phonated signal. We use the approach proposed in [31]—we estimate the VFO model parameters to minimize the error between the volume velocity of air predicted by the model and a reference signal representing the actual glottal excitation to the vocal tract. If the VFO model were to be considered in isolation, this reference could be obtained through actual physical measurements, e.g., through photography [5], physical or numerical simulations [7,17], or by inverse filtering the speech signal using a technique such as [35] (the approach used in [31]).

For the purpose of our discussion in this section, however, we do not specify where this reference excitation is obtained from, since the estimation of VFO model parameters from a given glottal excitation is only a waypoint towards estimation from the joint model that includes both the VFO and VT components. As we will see in Section 4.2, this does not in fact require explicit knowledge of the reference signal at the glottis.

Let $u_{g}\left(t\right)$ be the reference signal representing the *true* air-volume velocity at the glottis that excites the vocal tract. The volume velocity of air $u_{0}\left(t\right)$ (we remind the reader that $u_{0}\left(t\right)=u\left(0,t\right)$) at the glottis can also be computed from the vocal fold opening at the glottis as

(6) $$u_{0}\left(t\right)=\tilde{c}d(2\xi_{0}+\xi_{l}\left(t\right)+\xi_{r}\left(t\right))$$

where $\xi_{0}$ is the half glottal width at rest, $2\xi_{0}+\xi_{l}\left(t\right)+\xi_{r}\left(t\right)$ is the complete glottal opening, *d* is the length of the vocal fold, and $\tilde{c}$ is the air particle velocity at the midpoint of the vocal folds.

We assume that the movement of the vocal folds follows the VFO model of Section 3.1. Correspondingly, $\xi_{l}\left(t\right)$ and $\xi_{r}\left(t\right)$ must obey Equation (3), subject to boundary conditions. The model parameters α, β and Δ can hence be computed to minimize the difference between the air volume velocity $u_{0}\left(t\right)$ predicted by the model and the reference $u_{0}^{g}\left(t\right)$.

We define the instantaneous *residual R* as the error between $u_{0}\left(t\right)$ and $u_{g}\left(t\right)$:

(7) $$\begin{array}{cc} R(t) & =u_{0}\left(t\right)-u_{g}\left(t\right)\\ \; & =\tilde{c}d\left(2\xi_{0}+\xi_{l}\left(t\right)+\xi_{r}\left(t\right)\right)-u_{g}\left(t\right) \end{array}$$

The overall $\ell_{2}$ error between $u_{0}\left(t\right)$ and $u_{g}\left(t\right)$ is given by the integral

(8) $$F\left(\xi_{l},\xi_{r};\vartheta \right)=\int_{0}^{T}R^{2}\left(t\right)dt$$

where ϑ=[α,β,Δ] represents the parameters of the VFO model, and *T* represents the complete length of the reference signal.

The actual estimation can now be stated as

(9) $$\begin{array}{cc} \alpha^{*},\beta^{*},\Delta^{*} & =\arg\min_{\alpha ,\beta ,\Delta }F\left(\xi_{l},\xi_{r}\vartheta \right)\\ subject\;\;to \end{array}$$

(10) $$\begin{array}{cc} \; & {\ddot{\xi }}_{l}+\beta \left(1+\xi_{l}^{2}\right){\dot{\xi }}_{l}+\xi_{l}+\frac{\Delta }{2}\xi_{l}=\alpha \left({\dot{\xi }}_{r}+{\dot{\xi }}_{l}\right) \end{array}$$

(11) $$\begin{array}{cc} \; & {\ddot{\xi }}_{r}+\beta \left(1+\xi_{r}^{2}\right){\dot{\xi }}_{r}+\xi_{r}-\frac{\Delta }{2}\xi_{r}=\alpha \left({\dot{\xi }}_{r}+{\dot{\xi }}_{l}\right) \end{array}$$

(12) $$\begin{array}{cc} \; & \xi_{r}\left(0\right)=C_{r} \end{array}$$

(13) $$\begin{array}{cc} \; & \xi_{l}\left(0\right)=C_{l} \end{array}$$

(14) $$\begin{array}{cc} \; & {\dot{\xi }}_{r}\left(0\right)=0 \end{array}$$

(15) $$\begin{array}{cc} \; & {\dot{\xi }}_{l}\left(0\right)=0 \end{array}$$

where $C_{r}$ and $C_{l}$ are constants representing the quiescent positions of the vocal folds, and the folds are assumed to be at rest prior to the onset of phonation. For the computation we set $\xi_{0}$ to a typical value of 0.1 cm. The length of the vocal folds *d* may be set to 17.5 mm (which is within the range of normal lengths for both male and female subjects), and the air particle velocity $\tilde{c}$ to 5000 cm/s [2].

Note that given α,β and Δ the differential equations of model Equations (11)–(15) (the constraints) can be solved by any ODE solver to obtain $\xi_{l}\left(t\right)$ and $\xi_{r}\left(t\right)$. So, in principle, we could solve the constrained optimization problem of Equation (9)–(15) by a grid search over (α,β,Δ) to identify the specific values that minimize the squared error of Equation (8). This would, however, be highly inefficient.

Instead we propose the ADLES (“ADjoint LEast Squares”) algorithm, which restates the constraints (11)–(15) as Lagrangians on the objective, and derives a gradient descent solution. The detailed derivation of ADLES is given in Appendix C. We summarize the key steps below.

Incorporating constraints (11)–(15) into the objective, we define the Lagrangian:

(16) $$\begin{array}{cc} \; & L(\vartheta )=\\ \; & \int_{0}^{T}[R^{2}+\lambda \left({\ddot{\xi }}_{r}+\beta \left(1+\xi_{r}^{2}\right){\dot{\xi }}_{r}+\xi_{r}-\frac{\Delta }{2}\xi_{r}-\alpha \left({\dot{\xi }}_{r}+{\dot{\xi }}_{l}\right)\right)\\ \; & +\eta \left({\ddot{\xi }}_{l}+\beta \left(1+\xi_{l}^{2}\right){\dot{\xi }}_{l}+\xi_{l}+\frac{\Delta }{2}\xi_{l}-\alpha \left({\dot{\xi }}_{r}+{\dot{\xi }}_{l}\right)\right)]dt\\ \; & +\mu_{l}\left(\xi_{l}\left(0\right)-C_{l}\right)+\mu_{r}\left(\xi_{r}\left(0\right)-C_{r}\right)+\nu_{l}{\dot{\xi }}_{l}\left(0\right)+\nu_{r}{\dot{\xi }}_{r}\left(0\right) \end{array}$$

where λ, η, $\mu_{l},\mu_{r}$, $\nu_{l}$ and $\nu_{r}$ are Lagrangian multipliers. Note that λ and η are also functions of time (we have not explicitly shown the “(t)” above for brevity of notation).

We obtain the Lagrangian parameters λ and η as the solution to the following equations: For 0<t<T:

(17) $$\begin{array}{ccc} \ddot{\lambda }+\left(2\beta \xi_{r}{\dot{\xi }}_{r}+1-\frac{\Delta }{2}\right)\lambda +2\tilde{c}dR & =0 & \; \end{array}$$

(18) $$\begin{array}{ccc} \ddot{\eta }+\left(2\beta \xi_{l}{\dot{\xi }}_{l}+1+\frac{\Delta }{2}\right)\eta +2\tilde{c}dR & =0 & \; \end{array}$$

(19) $$\begin{array}{ccc} \beta \left(1+\xi_{r}^{2}\right)\lambda -\alpha \left(\lambda +\eta \right) & =0 & \; \end{array}$$

(20) $$\begin{array}{ccc} \beta \left(1+\xi_{l}^{2}\right)\eta -\alpha \left(\lambda +\eta \right) & =0 & \; \end{array}$$

with initial conditions at t=T:

(21) $$\begin{array}{cc} \lambda (T) & =0 \end{array}$$

(22) $$\begin{array}{cc} \dot{\lambda }\left(T\right) & =0 \end{array}$$

(23) $$\begin{array}{cc} \eta (T) & =0 \end{array}$$

(24) $$\begin{array}{cc} \dot{\eta }\left(T\right) & =0 \end{array}$$

Note that given α, β, Δ, $\xi_{r}$ and $\xi_{l}$, Equations (17)–(24) represent a differential-algebraic system of equations and can be solved by any DAE solver to obtain λ and η.

Given $\xi_{r}$, $\xi_{l}$, λ and η, the derivatives of $F(\xi_{l},\xi_{r},\vartheta )$ w.r.t. α, β and Δ can now be obtained as:

(25) $$\begin{array}{cc} F_{\alpha } & =\int_{0}^{T}-\left({\dot{\xi }}_{r}+{\dot{\xi }}_{l}\right)\left(\lambda +\eta \right)dt\\ F_{\beta } & =\int_{0}^{T}\left(\left(1+\xi_{r}^{2}\right){\dot{\xi }}_{r}\lambda +\left(1+\xi_{l}^{2}\right){\dot{\xi }}_{l}\eta \right)dt\\ F_{\Delta } & =\int_{0}^{T}\frac{1}{2}\left(\xi_{l}\eta -\xi_{r}\lambda \right)dt \end{array}$$

The derivatives from Equation (25) are plugged into a gradient descent update rule for the model parameters:

(26) $$\begin{array}{c} \alpha ⟵\alpha -\tau^{\alpha }F_{\alpha }\\ \beta ⟵\beta -\tau^{\beta }F_{\beta }\\ \Delta ⟵\Delta -\tau^{\Delta }F_{\Delta } \end{array}$$

where $\tau^{\cdot }$ is the step-size.

The overall ADLES algorithm is summarized in Algorithm 1:
**Algorithm 1** ADLES algorithm1:Initialize α, β and Δ2:**while** *F* not converged **do**                                ▹* Iterate until the error converges*3:    Solve (11)–(10) with initial conditions (12)–(15), using the current estimates of α, β and Δ, obtaining $\xi_{r}$, $\xi_{l}$, ${\dot{\xi }}_{r}$ and ${\dot{\xi }}_{l}$.4:    Solve (17)–(20) with the initial conditions (21)–(24), using the current values of $\xi_{l}$, $\xi_{r}$, α, β and Δ, obtaining λ, $\dot{\lambda }$, η and $\dot{\eta }$.5:    Compute $F_{\alpha }$, $F_{\beta }$ and $F_{\Delta }$ from Equation (25).6:    Update α, β and Δ with (26).7:**end while**

### 4.2. Estimating VFO Parameters from the Speech Signal: The Forward-Backward Approach (ADLES-VFT)

The backward approach, solved by the ADLES algorithm in Section 4.1, derives the VFO parameters by minimizing the error between the output of the VFO model $u_{0}\left(t\right)$ and the glottal excitation $u_{g}\left(t\right)$. However, in general, $u_{g}\left(t\right)$ is not available, and this error cannot actually be computed.

Instead, in the forward-backward approach, we further *propagate* the generated excitation $u_{0}\left(t\right)$ through the vocal tract, represented by the VT model of Equation (4) to obtain a signal $u_{L}\left(t\right)=u\left(L,t\right)$ at the lips. This is the output of the joint VFO and VT models. We compute the error between the generated signal $u_{L}\left(t\right)$ and the air velocity measurement derived from the recorded voice signal, which *is* available, and propagate this error backward through the vocal tract, to obtain the error at the glottis. The VT and VFO model parameters are estimated to minimize this error. Thus, the algorithm itself proceeds through iterations of two steps: a forward step in which the VFO-generated excitation is propagated through the VT model to generate an output signal, and a backward step in which the error between the generated signal and the recorded speech is propagated backward through the VT to adjust the model parameters. We explain the entire procedure below.

The recorded voice signal is, in fact, a pressure wave and records the pressure wave emitted at the lips. Let $p_{m}\left(t\right)$ be the measured acoustic pressure at the lip. The corresponding volume velocity is given by [34]

(27) $$u_{m}\left(t\right)=\frac{A(L)}{\rho c}p_{m}\left(t\right)$$

$u_{m}\left(t\right)$ is now our reference signal at the lips to which $u_{L}\left(t\right)$ must be matched, in order to estimate model parameters.

The propagation of $u_{0}\left(t\right)=u\left(0,t\right)$ through the vocal tract is assumed to follow the dynamics of Equation (4). Let $H_{f}$ be the nonlinear operator representing acoustic wave propagation through the vocal tract from the glottis to the lip. The subscript *f* in $H_{f}$ represents the vocal-tract profile f(x,t) in Equation (4) and indicates the dependence of $H_{f}$ on f(x,t). Thus the vocal-tract output $u_{L}\left(t\right)$ is given by

(28) $$u_{L}\left(t\right)=H_{f}\left(u_{0}\left(t\right)\right)=H_{f}\left(\tilde{c}d\left(2\xi_{0}+\xi_{l}\left(t\right)+\xi_{r}\left(t\right)\right)\right)$$

Our objective is to minimize the difference between the measured volume velocity $u_{m}\left(t\right)$ and the predicted volume velocity $u_{L}\left(t\right)$ near the lip subject to constraint that the dynamics of the vocal folds must follow the VFO model of Equation (3). Note that the parameters of the joint model include the VFO model parameters α, β and Δ, and the vocal tract profile f(x,t) required by the VT model. Although we only require the VFO model parameters to determine vocal fold oscillation, the minimization must be performed against all of these. Thus the estimation problem becomes

(29) $$\begin{array}{cc} \; & \min\int_{0}^{T}{\left(u_{L}\left(t\right)-u_{m}\left(t\right)\right)}^{2}dt\\ \Leftrightarrow \; & \min\int_{0}^{T}{\left(u_{L}\left(t\right)-\frac{A(L)}{\rho c}p_{m}\left(t\right)\right)}^{2}dt \end{array}$$

subject to

(30) $$\begin{array}{cc} \; & {\ddot{\xi }}_{r}+\beta \left(1+\xi_{r}^{2}\right){\dot{\xi }}_{r}+\xi_{r}-\frac{\Delta }{2}\xi_{r}=\alpha \left({\dot{\xi }}_{r}+{\dot{\xi }}_{l}\right) \end{array}$$

(31) $$\begin{array}{cc} \; & {\ddot{\xi }}_{l}+\beta \left(1+\xi_{l}^{2}\right){\dot{\xi }}_{l}+\xi_{l}+\frac{\Delta }{2}\xi_{l}=\alpha \left({\dot{\xi }}_{r}+{\dot{\xi }}_{l}\right) \end{array}$$

(32) $$\begin{array}{cc} \; & \;\left(I.C.1\right)\;\xi_{r}\left(0\right)=C_{r} \end{array}$$

(33) $$\begin{array}{cc} \; & \;\left(I.C.2\right)\;\xi_{l}\left(0\right)=C_{l} \end{array}$$

(34) $$\begin{array}{cc} \; & \;\left(I.C.3\right)\;{\dot{\xi }}_{r}\left(0\right)=0 \end{array}$$

(35) $$\begin{array}{cc} \; & \;\left(I.C.4\right)\;{\dot{\xi }}_{l}\left(0\right)=0 \end{array}$$

where, as before, (30) and (31) represent the asymmetric vocal folds displacement model (3), I.C. stands for initial condition, and *C*s are constants. The minimization is performed against the complete set of parameters of the joint VFO-VT model, i.e., α, β, Δ and f(x,t).

Unlike in Equations (11)–(15), this cannot be solved, even in principle, by simply scanning for the optimal α, β and Δ, since $H_{f}$ is characterized by f(x,t) which is also unknown and must be determined.

To solve the optimization problem of (29)–(35), we derive an efficient gradient-descent solution which we term the ADLES-VFT algorithm. The essential steps of the solution are given below. The details of the derivation are in Appendix D.

#### 4.2.1. Forward Pass

First, note that, as before, the constraint Equations (30)–(35) are ordinary differential equations with initial conditions that, given α, β and Δ, can be solved by any ODE solver. The solution will give us the VFO model generated glottal excitation $u_{0}\left(t\right)$.

Next, we propagate the generated excitation $u_{0}\left(t\right)$ through the VT model. For this, we must solve

(36) $$\begin{array}{cc} \; & \frac{\partial^{2}u\left(x,t\right)}{\partial t^{2}}=c^{2}\frac{\partial^{2}u\left(x,t\right)}{\partial x^{2}}+f\left(x,t\right)\\ \;\mathrm{subject}\;\mathrm{to}\;\; & \end{array}$$

(37) $$\begin{array}{cc} \;\left(B.C.1\right)\;\; & u\left(0,t\right)=u_{0}\left(t\right) \end{array}$$

(38) $$\begin{array}{cc} \;\left(B.C.2\right)\;\; & \frac{\;\;\partial u}{\partial n_{\Gamma }}=0 \end{array}$$

(39) $$\begin{array}{cc} \;\left(I.C.1\right)\;\; & \;u(x,0)=0 \end{array}$$

(40) $$\begin{array}{cc} \;\left(I.C.2\right)\;\; & \frac{\partial u(x,0)}{\partial t}=0 \end{array}$$

where B.C. stands for boundary condition, and I.C. stands for initial condition. The vocal tract is assumed to be circular at the glottis and the lips. Here, $n_{\Gamma }$ is the outward unit normal to the vocal tract boundary Γ, at the glottis.

Equations (36)–(40) represent a set of partial differential equations. The boundary conditions relate to the air volume velocity at the glottal end of the vocal tract. The initial conditions relate to air volume velocity at the initial time, t=0, when the generated glottal signal $u_{0}\left(t\right)$ enters the vocal tract. Given $u_{0}\left(t\right)$ and f(x,t) (36)–(40) can be solved using a PDE solver. In our work we use the finite-element method described in Appendix E.

Solving (36)–(40) gives us u(x,t) at all positions x∈(0,L) and time t∈(0,T). In the process, it also gives us $H_{f}\left(u_{0}\left(t\right)\right)=u_{L}\left(t\right)=u\left(L,t\right)$.

#### 4.2.2. Backward Pass

The backward pass updates all model parameters including the VT term f(x,t), and VFO parameters based on the error at the output.

Updating *f*:

We denote the estimation residual as:

(41) $$r\left(t\right)=u_{m}\left(t\right)-H_{f}\left(u_{0}\left(t\right)\right)$$

We must propagate this residual backward through the VT model. To do so, we use a time reversal technique [36] and backpropagate the difference (41) into the vocal tract, which gives:

(42) $$\begin{array}{cc} \; & \frac{\partial^{2}z}{\partial t^{2}}=c^{2}\frac{\partial^{2}z}{\partial x^{2}}+f\left(x,t\right)\\ \;\mathrm{subject}\;\mathrm{to}\;\; & \end{array}$$

(43) $$\begin{array}{cc} \;\left(B.C.1\right)\;\; & \;\;\frac{\partial z}{\partial n_{\Gamma }}=r \end{array}$$

(44) $$\begin{array}{cc} \;\left(I.C.1\right)\;\; & z\left(x,T\right)=0 \end{array}$$

(45) $$\begin{array}{cc} \;\left(I.C.2\right)\;\; & \frac{\partial z\left(x,T\right)}{\partial t}=0 \end{array}$$

where *z* is the time reversal of *u*. Note that the boundary conditions and initial conditions in (42)–(45) are now defined at the lip, and the equation itself propagates backward through the vocal tract.

As before, Equations (42)–(45) can be solved by the finite-element method of Appendix E to give us z(x,t). The gradient update rule for f(x,t) is then obtained as

(46) $$f⟵f+\iota \left(\frac{z}{c^{2}}+u\right).$$

where ι is a learning rate parameter (see Appendix D).

Updating α, β, Δ:

As in the case of the backward approach of Section 4.1 we define a residual

(47) $$R\left(t\right)=u_{L}\left(t\right)-u_{m}\left(t\right)=H_{f}\left(\tilde{c}d\left(2\xi_{0}+\xi_{l}\left(t\right)+\xi_{r}\left(t\right)\right)\right)-\frac{A(L)}{\rho c}p_{m}\left(t\right)$$

Note that unlike in Section 4.1 the residual in Equation (47) is defined at the lips, rather than at the glottis. As before, we can define the total squared residual error as

(48) $$F\left(\xi_{l},\xi_{r};\vartheta \right)=\int_{0}^{T}R^{2}\left(t\right)dt$$

where ϑ=[α,β,Δ] are the parameters of the vocal folds model (3). *F* must be minimized with respect to ϑ, subject to the constraints imposed by the VFO model.

Once again, as in Section 4.1 we fold in the constraints into the objective through Lagrangian multipliers as

(49) $$\begin{array}{cc} L(\vartheta )= & \int_{0}^{T}\left[R^{2}+\lambda \left({\ddot{\xi }}_{r}+\beta \left(1+\xi_{r}^{2}\right){\dot{\xi }}_{r}+\xi_{r}-\frac{\Delta }{2}\xi_{r}-\alpha \left({\dot{\xi }}_{r}+{\dot{\xi }}_{l}\right)\right)\right.\\ \; & \left.+\eta \left({\ddot{\xi }}_{l}+\beta \left(1+\xi_{l}^{2}\right){\dot{\xi }}_{l}+\xi_{l}+\frac{\Delta }{2}\xi_{l}-\alpha \left({\dot{\xi }}_{r}+{\dot{\xi }}_{l}\right)\right)\right]dt\\ \; & +\mu_{l}\left(\xi_{l}\left(0\right)-C_{l}\right)+\mu_{r}\left(\xi_{r}\left(0\right)-C_{r}\right)+\nu_{l}{\dot{\xi }}_{l}\left(0\right)+\nu_{r}{\dot{\xi }}_{r}\left(0\right) \end{array}$$

where λ, η, μ and ν are multipliers, and λ and η are themselves functions of time. Optimization requires minimization of L(ϑ) with respect to α, β and Δ.

This leads us (Appendix D) to the following set of equations for λ and η:

(50) $$\begin{array}{cc} \; & \ddot{\lambda }+\left(2\beta \xi_{r}{\dot{\xi }}_{r}+1-\frac{\Delta }{2}\right)\lambda +2\tilde{c}dR\frac{u_{L}}{u_{0}}=0 \end{array}$$

(51) $$\begin{array}{cc} \; & \ddot{\eta }+\left(2\beta \xi_{l}{\dot{\xi }}_{l}+1+\frac{\Delta }{2}\right)\eta +2\tilde{c}dR\frac{u_{L}}{u_{0}}=0 \end{array}$$

(52) $$\begin{array}{cc} \; & \beta \left(1+\xi_{r}^{2}\right)\lambda -\alpha \left(\lambda +\eta \right)=0 \end{array}$$

(53) $$\begin{array}{cc} \; & \beta \left(1+\xi_{l}^{2}\right)\eta -\alpha \left(\lambda +\eta \right)=0 \end{array}$$

with initial conditions (at t=T, i.e., at the lips):

(54) $$\begin{array}{cc} \; & \lambda \left(T\right)=0 \end{array}$$

(55) $$\begin{array}{cc} \; & \dot{\lambda }\left(T\right)=0 \end{array}$$

(56) $$\begin{array}{cc} \; & \eta \left(T\right)=0 \end{array}$$

(57) $$\begin{array}{cc} \; & \dot{\eta }\left(T\right)=0 \end{array}$$

Given R(t), $u_{0}\left(t\right)$ and $u_{L}\left(t\right)$ Equations (50)–(57) represent a set of differential-algebraic equations and can be solved with a DAE solver.

We finally obtain the derivative of *F* w.r.t. α, β and Δ (represented below as $F_{\alpha }$, $F_{\beta }$ and $F_{\Delta }$, respectively) as

(58) $$\begin{array}{cc} F_{\alpha } & =\int_{0}^{T}-\left({\dot{\xi }}_{r}+{\dot{\xi }}_{l}\right)\left(\lambda +\eta \right)dt \end{array}$$

(59) $$\begin{array}{cc} F_{\beta } & =\int_{0}^{T}\left(\left(1+\xi_{r}^{2}\right){\dot{\xi }}_{r}\lambda +\left(1+\xi_{l}^{2}\right){\dot{\xi }}_{l}\eta \right)dt \end{array}$$

(60) $$\begin{array}{cc} F_{\Delta } & =\int_{0}^{T}\frac{1}{2}\left(\xi_{l}\eta -\xi_{r}\lambda \right)dt \end{array}$$

The gradient descent update rules for the VFO model parameters are finally obtained as

(61) $$\begin{array}{cc} \alpha & ⟵\alpha -\tau^{\alpha }F_{\alpha } \end{array}$$

(62) $$\begin{array}{cc} \beta & ⟵\beta -\tau^{\beta }F_{\beta } \end{array}$$

(63) $$\begin{array}{cc} \Delta & ⟵\Delta -\tau^{\Delta }F_{\Delta } \end{array}$$

#### 4.2.3. The ADLES-VFT Algorithm Summarized

The overall ADLES-VFT algorithm for solving the parameter estimation problem (29)–(35) is summarized in Algorithm 2.

In this solution, we have adopted the simple gradient descent method. However, other gradient-based optimization approaches, such as the *conjugate gradient* method, can also be used.

We note here that Algorithm 2 requires several terms to be specified. In our implementation, the quiescent positions of the vocal folds, $C_{r}$ and $C_{l}$ were set to 0. We initialize [α,β,Δ]=[0.8,0.32,1.0]—these values were empirically found to work best. f(x,t) is initialized to 0. This effectively initializes Equation (4) to the Webster Horn equation. The step sizes $\tau^{\alpha },\tau^{\beta }$ and $\tau^{\Delta }$ are all adaptively set to $0.01/max(F_{\alpha },F_{\beta },F_{\Delta })$, and ι is set to 1. The actual objective minimize, Equation (29), requires scaling $p_{m}\left(t\right)$ by A(L)/ρc prior to comparison to $u_{L}\left(t\right)$. In practice, since $p_{m}\left(t\right)$ is derived from a voice signal recorded at a distance from the lips, the unknown transmission loss between the lips and the microphone must also be considered. To deal with this, we simply normalize both sequences to 0 mean and unit variance, and do not apply any additional scaling.
**Algorithm 2** ADLES-VFT algorithm1:Initialize α, β, Δ and f(x,t).2:**while** *F* not converged **do**                                                        ▹ *Iterate until the error converges*3:    Solve (30)–(31) with initial conditions (32)–(35) with an ODE solver, using the current estimates of α, β and Δ, obtaining $\xi_{r}$, $\xi_{l}$, ${\dot{\xi }}_{r}$ and ${\dot{\xi }}_{l}$ and $u_{0}\left(t\right)$.4:    Using current estimate of f(x,t) and $u_{0}\left(t\right)$, solve the forward propagation model (36)–(40) for $u_{L}\left(t\right)$ with a PDE solver, e.g., the finite-element method of Appendix E.5:    Calculate the estimation difference r(t) using (41).6:    Using the current estimate of f(x,t) and r(t), solve the backward propagation model (42)–(45) for z(x,t) with a PDE solver (Appendix E).7:    Update f(x,t) using (46).8:    Solve (50)–(53) with initial conditions (54)–(57) using a DAE solver to obtain λ, $\dot{\lambda }$, η and $\dot{\eta }$.9:    Compute (58)–(60) through numerical integration to obtain derivatives $F_{\alpha }$, $F_{\beta }$ and $F_{\Delta }$ from10:    Update α, β and Δ with (63).11:**end while**

In the next section, we demonstrate the usefulness of the ADLES and ADLES-VFT algorithms experimentally.

## 5. Experimental Results and Interpretation

Unfortunately, until the time of writing this paper, we could not obtain actual electroglottographic measurements or video data of vocal fold motion to compare our derived VFOs to. However, from a computational perspective the algorithms proposed can still be validated in different ways. We explain these below.

### 5.1. Validation 1

Our first validation approach is to use the proxy of showing that the solutions obtained are indeed discriminative of fine-level changes in glottal flow dynamics of the phonation process.

Having recovered the model parameters by our backward or forward approach, we can solve the models to obtain the time-series corresponding to the oscillations of each vocal fold, as estimated from recorded speech samples. We note that the models we have discussed in this paper are essentially dynamical systems represented by coupled nonlinear equations that may not have closed-form solutions, but can be numerically solved.

To interpret these in discriminative settings, we can utilize some well-established methods for characterizing dynamical systems, borrowing them from chaos theory and other areas of applied mathematics (e.g., flow, orbit, attractor, stability, Poincaré map, bifurcation, Lyapunov exponents, etc.). These are described in Appendix F.

#### Interpreting a System’s Phase Portraits Using Its Bifurcation Map

Appendix F describes the concepts and tools used to study the behaviors (e.g., flow, orbit, attractor, stability, Poincaré map, bifurcation) of nonlinear dynamical systems such as Equation (3). The phase space of the system in Equation (3) (representing vocal fold motion) is four-dimensional and includes states $\left(\xi_{r},{\dot{\xi }}_{r},\xi_{l},{\dot{\xi }}_{l}\right)$.

For this nonlinear system, it is expected that attractors such as limit cycles or toruses will appear in the phase space. Such phenomena are consequences of specific parameter settings. Specifically, the parameter β determines the periodicity of oscillations; the parameter α and Δ quantify the asymmetry of the displacement of left and right vocal folds and the degree to which one of the vocal folds is out of phase with the other [24,37]. We can visualize them by plotting the left and right displacements and the phase space portrait.

**Figure 5:**
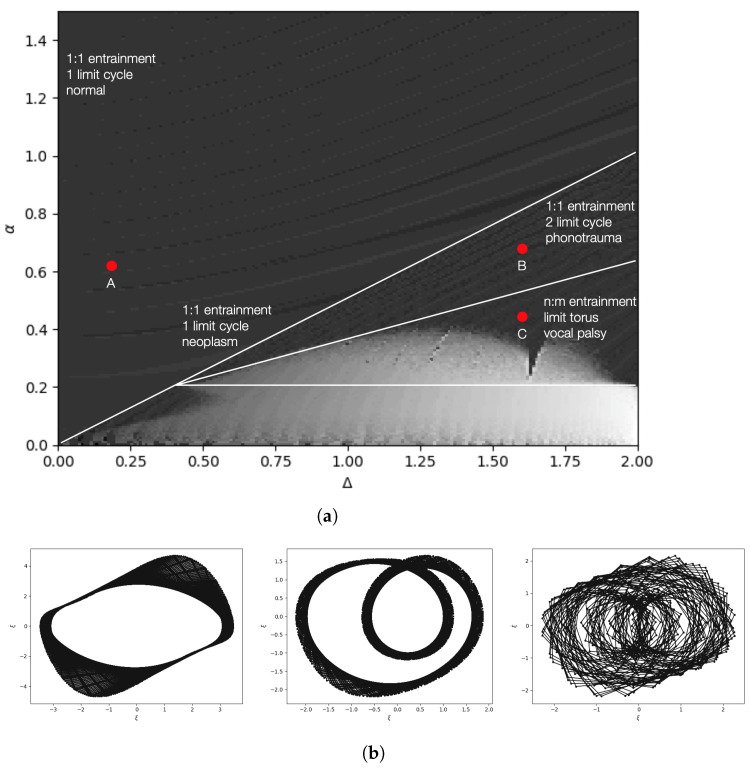
(**a**) A 3D Bifurcation diagram of the asymmetric vocal fold model. The third dimension is perpendicular to the parameter plane shown, and depicts the entrainment ratio n:m (encoded in different shades of gray) as a function of model parameters α and Δ, where *n* and *m* are the number of intersections of the orbits of right and left oscillators across the Poincaré section ${\dot{\xi }}_{r,l}=0$ at stable status. This is consistent with the theoretical results in [24]); (**b**) Phase-space trajectories (or phase portraits) corresponding to the points A (left panel), B (center panel) and C (right panel). The horizontal axis is displacement of a vocal fold, and the vertical axis is its velocity.

The coupling of right and left oscillators is described by their *entrainment*; they are in *n*:*m* entrainment if their phases $\theta_{r}$, $\theta_{l}$ satisfy $|n\theta_{r}-m\theta_{l}|<C$ where n,m are integers, and *C* is a constant [24]. Such entrainment can be revealed by the Poincaré map, where the number of trajectory crossings of the right or left oscillators within the Poincaré section indicates the periodicity of its limit cycles. Therefore, their ratio represents the entrainment. We can use the bifurcation diagram to visualize how the entrainment changes with parameters. An example of such a bifurcation diagram is shown in Figure 5 [15,37].

As we will see later (and as indicated in Figure 5), model parameters can characterize voice pathologies, and these can be visually evident in in phase portraits and bifurcation plots.

We use the backward algorithm to estimate the asymmetric model parameters for clinically acquired pathological speech data. The data comprise speech samples collected from subjects suffering from three different vocal pathologies. Our goal is to demonstrate that the individualized phase space trajectories of the asymmetric vocal fold model are discriminative of these disorders.

The data used in our experiments is the FEMH database [38]. It comprises 200 recordings of the sustained vowel /a:/. The data were obtained from a voice clinic in a tertiary teaching hospital, and the complete database includes 50 normal voice samples (control set) and 150 samples that represent common voice pathologies. Specifically, the set contains 40/60/50 samples for glottis neoplasm, phonotrauma (including vocal nodules, polyps, and cysts), and unilateral vocal paralysis, respectively.

**Figure 6:**
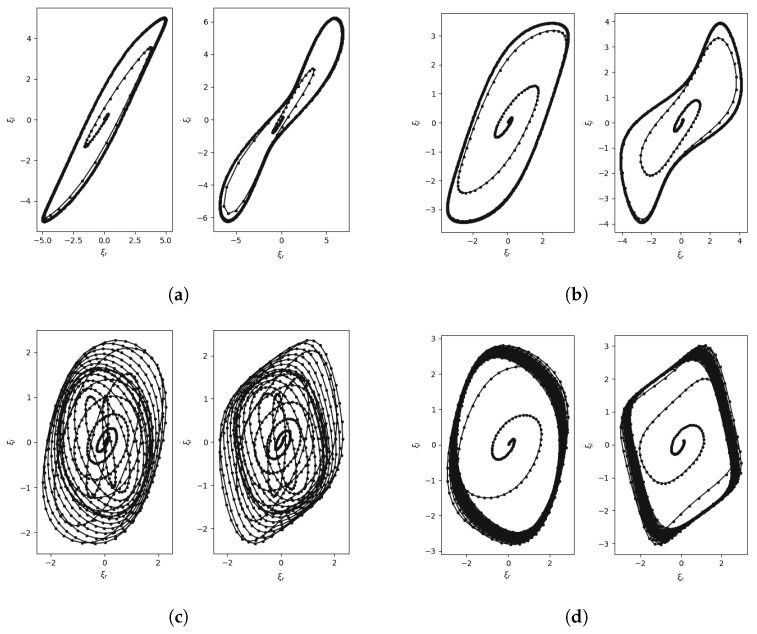
Phase portraits showing the coupling of the left and right oscillators (ADLES-based estimation) for (**a**) normal speech: 1 limit cycle, (**b**) neoplasm: 1 limit cycle, (**c**) phonotrauma: 2 limit cycles, (**d**) vocal palsy: limit torus. The convergence trajectory is also shown, and the limit cycles can be observed as the emergent geometries in these plots.

Figure 6 shows some phase portraits showing the coupling of the right and left vocal folds obtained using the ADLES solution. We observe that the attractor behaviors are typical and even visually differentiable for different types of pathologies.

Table 1 shows the results of deducing voice pathologies by simple thresholding of parameter ranges. Specifically, the ranges of model parameters in each row of Table 1 correspond to regions in the bifurcation diagram in Figure 5. Each region has distinctive attractors and phase entrainment, representing distinct vocal fold behaviors and thereby indicating different voice pathologies. By extracting the phase trajectories for the speech signal and, thereby, the underlying system parameters, the ADLES algorithm can place the vocal-fold oscillations in voice production on the bifurcation diagram and thus deduce the pathology.

### 5.2. Validation 2

Our second validation approach is to compare the excitation signal obtained through inverse filtering with the glottal flow signal (VFO) obtained through the backward or forward-backward algorithm. The rationale behind this is that within reasonable bounds of error, the glottal flow signal obtained through our model is expected to conform to the oscillation patterns seen in the excitation signal for each speaker.

**Figure 7:**
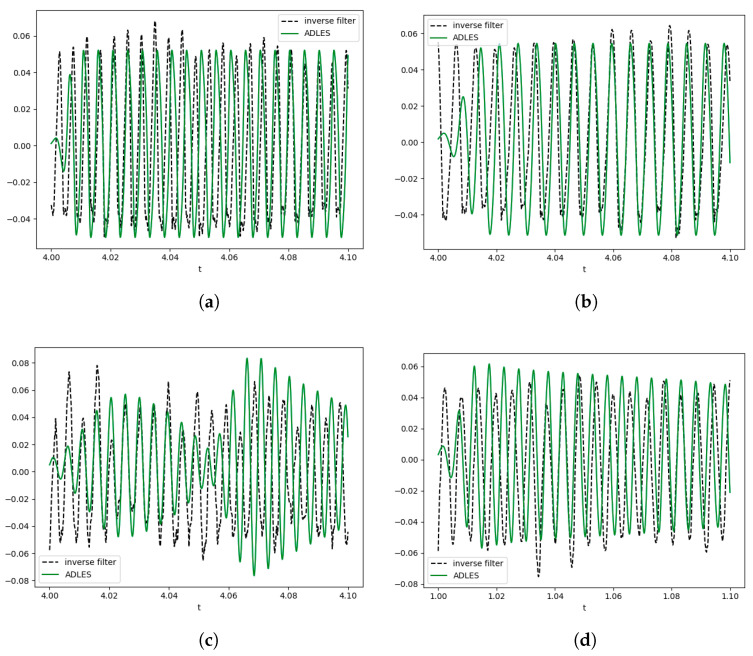
Glottal flows from inverse filtering and ADLES estimation for (**a**) normal speech (control), (**b**) neoplasm, (**c**) phonotrauma, and (**d**) vocal palsy.

Figure 7 shows the glottal flow obtained by inverse filtering and those obtained by the asymmetric model with the parameters estimated by our ADLES method. We observe consistent matches, showing that the ADLES algorithm does achieve its objectives in individualizing the asymmetric model to each speaker instance.

### 5.3. Validation 3

Our third validation approach is to compare the estimation precision of the backward approach and the forward-backward approach. Table 2 shows the mean absolute error (MAE) of calculating glottal flows and parameters for four voice types (normal, neoplasm, phonotrauma, vocal palsy) obtained by backward (ADLES) and forward-backward (ADLES-VFT) algorithms. The glottal flows obtained by inverse filtering the speech signals are treated as ground truths. Since there is no ground truth for model parameters, we treat the parameters obtained by ADLES as ground truth. These results suggest that our forward-backward algorithm can effectively recover the vocal tract profile, glottal flow, and model parameters.

### 5.4. Validation 4

Our fourth validation comes indirectly from prior studies. Information from a dynamical systems perspective can give insights about the underlying mechanisms and principles that govern the vocal fold dynamics. Examples of features in this category are recurrence analysis features, Lyapunov exponents, Hurst exponents, etc. These are mentioned in Appendix F.

Some of these features have been used in real-world applications and proven to be effective. For example, in [39], the authors hypothesize that since COVID-19 impairs the respiratory system, effects on the phonation process could be expected, and signatures of COVID-19 could manifest in the vibration patterns of the vocal folds. In this paper, features have been derived from a signal processing perspective.

This study used the ADLES method to estimate the asymmetric vocal folds model parameters. It further used the parameters and estimation residuals as features to other binary classifiers such as logistic regression, support vector machine, decision tree, and random forest, achieving around 0.8 ROC-AUC (area under the ROC curve) in discriminating positive COVID-19 cases from negative instances, on clinically collected and curated data. The data used contained recordings of extended vowel sounds from affected speakers and control subjects. The authors also discovered that COVID-19 positive individuals display different phase space behaviors from negative individuals: the phase space trajectories for negative individuals were found to be more regular and symmetric across the two vocal folds, while the trajectories for positive patients were more chaotic, implying a lack of synchronization and a higher degree of asymmetry in the vibrations of the left and right vocal folds.

In a companion study, the authors in [40] used the ADLES-estimated glottal flows as features to CNN-based two-step attention neural networks. The neural model detects differences in the estimated and actual glottal flows and predicts two classes corresponding to COVID-19 positive and negative cases. This achieved 0.9 ROC-AUC (normalized) on clinically collected vowel sounds. Yet another study used higher order statistics derived from parameters, and Lyapunov and Hurst exponents derived from the phase space trajectories of the individualized asymmetric models, to detect Amyotrophic Lateral Sclerosis (ALS) from voice with high accuracy (normalized ROC-AUC of 0.82 to 0.99) [41].

## 6. Conclusions and Future Directions

In this paper we have presented a dynamical system perspective for physical process modeling and phase space characterization of phonation, and proposed a framework wherein these can be derived for individual speakers from recorded speech samples. The oscillatory dynamics of vocal folds provide a tool to analyze different phonation phenomena in many real-world task settings. We have proposed a backward approach for modeling vocal fold dynamics, and an efficient algorithm (the ADLES algorithm) to solve the inverse problem of estimating model parameters from speech observations. Further, we have integrated the vocal tract and vocal folds models, and have presented a forward-backward paradigm (the ADLES-VFT algorithm) for effectively solving the inverse problem for the coupled vocal fold-tract model. Extensions of these approaches can use other physical models of voice production, and other physical processes including phonation.

We have shown that the parameters estimated by these algorithms allow the models to closely emulate the vocal fold motion of individual speakers. Features and statistics derived from the model dynamics are (at least) discriminative enough for use in regular machine-learning based classification algorithms to accurately identify various voice pathologies from recorded speech samples. In future, these approaches are expected to be helpful in deducing many other underlying influences on the speaker’s vocal production mechanism.

The phase space characterization presented in this paper is based on phase space trajectories (a topological perspective)—the left and right vocal fold oscillations, velocities or accelerations. Measurements can also be performed from statistical, signal processing, information-theoretic and other perspectives. Another direction of suggested research is characterizing the phase space from algebraic perspectives. We can recast the study of the topological structures of the phase space to the study of its algebraic constructs, such as homotopy groups and homology/cohomology groups, which are easier to classify. For example, algebraic invariants can characterize the homeomorphisms between phase spaces (e.g., evolution maps, Poincaré maps) and reveal large-scale structures and global properties (e.g., existence and structure of orbits). We can also build upon the deep connection between dynamical systems and deep neural models. We can study deep learning approaches for solving and analyzing dynamical systems, and explore the integration of dynamical systems with deep neural models to analyze and interpret the behaviors of the vocal folds. We delegate these explorations to future work.

## Figures and Tables

**Table 1:** Parameters obtained and pathologies identified through ADLES.

Δ	α	Phase Space Behavior	Pathology	Accuracy
<0.5	>0.25	1 limit cycle, 1:1 entrain	Normal	0.90
∼0.6	∼0.35	1 limit cycle, 1:1 entrain	Neoplasm	0.82
∼0.6	∼0.3	2 limit cycles, 1:1 entrain	Phonotrauma	0.95
∼0.85	∼0.4	toroidal, *n*:*m* entrain	Vocal Palsy	0.89

**Table 2:** Estimation error by backward and forward-backward approach.

	Glottal Flow MAE		Parameter MAE	
	ADLES-B	ADLES-VFT	α	Δ
Normal	0.021	0.022	0.042	0.049
Neoplasm	0.028	0.036	0.055	0.058
Phonotrauma	0.043	0.051	0.083	0.079
Vocal palsy	0.059	0.065	0.102	0.119
All	0.040	0.045	0.074	0.078

**Figure A1:**
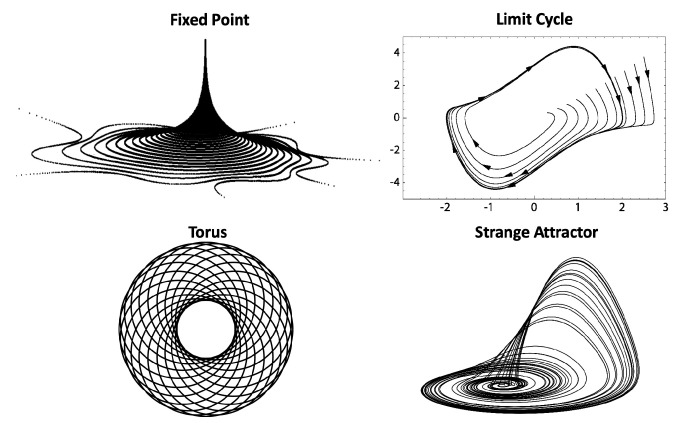
Illustration of different attractors in a dynamical system.

## Data Availability

This study used the Far Eastern Memorial Hospital (FEMH) database, which is available to all participants through the annual IEEE FEMH Voice Data Challenges. A similar dataset called “VOICED Database” is openly available through PhysioNet at: https://physionet.org/content/voiced/1.0.0/ (accessed on 1 June 2023).

## Appendix A

The following is a brief description of various VFO models:

- **Two-mass models**:

Two-mass models describe vocal fold motion as two coupled mass-damper-spring oscillators:

(A1) $$\begin{array}{cc} M_{1}{\ddot{x}}_{1}+B_{1}{\dot{x}}_{1}+K\left(x_{1}-x_{2}\right)+R_{1} & =F_{1} \end{array}$$

(A2) $$\begin{array}{cc} M_{2}{\ddot{x}}_{2}+B_{2}{\dot{x}}_{2}+K\left(x_{2}-x_{1}\right)+R_{2} & =F_{2} \end{array}$$

where $x_{i}$, $M_{i}$, $B_{i}$ are the *i*-th oscillator’s displacement, mass, and viscous damping coefficient, *K* is the coupling stiffness between the two masses, $F_{i}$ is the driving force, and $R_{i}$ is the elastic restoring force [15]. This model assumes (1) small air inertia and quasi-steady glottal flow, (2) negligible supra-glottal pressure, and (3) that the nonlinearity induced by vocal fold collision is small. These assumptions lead to small-amplitude oscillations and model simplification.

- **Multi-mass models**:
  Multi-mass models have a greater degrees of freedom and hence can model vocal fold motion with high precision. They are based on mass-spring-damper motion dynamics which are widely used in multiple problem settings (e.g., [42]). For the *i*-th mass component, the equation of motion is:
  (A3) $$M_{i}{\ddot{x}}_{i}=F_{i}^{A}+F_{i}^{V}+F_{i}^{L}+F_{i}^{C}+F_{i}^{D}$$
  where $x_{i}=\left(x_{i},y_{i},z_{i}\right)$ is the three-dimensional displacement, $M_{i}$ is the mass, $F_{i}^{A}$ is the anchor force associated with the anchor spring and damper, $F_{i}^{V}$ and $F_{i}^{L}$ are the vertical and longitudinal coupling forces associated with spring and damping, $F_{i}^{C}$ is the collision restoring force, and $F_{i}^{D}$ is the driving force from glottal pressure [18]. In [18], 50 masses are used.

- **Finite element models**:
  Finite element models discretize the vocal fold motion in space and time—the geometry of the vocal fold is discretized into small elements (cells). In each cell, the applicable differential equation governed by the law of physics is solved. These models can handle complex geometries, continuous deformation, and complex driving forces [17].
  Consider a cube element with six stress and strain components. By the principles of elasticity in mechanics we have:
  (A4) σ=Sϵ
  where σ is the stress tensor, ϵ is the strain tensor, and S is the stiffness matrix consisting of Young’s modulus, shear modulus, and Poisson’s ratio [17]. The relation between stress and displacement is governed by:
  (A5) $$\begin{array}{cc} \sigma_{x} & =C_{1}\mu \frac{\partial u}{\partial x}+C_{2}\mu \frac{\partial w}{\partial z} \end{array}$$
  (A6) $$\begin{array}{cc} \sigma_{z} & =C_{2}\mu \frac{\partial u}{\partial x}+C_{1}\mu \frac{\partial w}{\partial z} \end{array}$$
  (A7) $$\begin{array}{cc} \tau_{xy} & =\mu^{\prime }\frac{\partial u}{\partial y} \end{array}$$
  (A8) $$\begin{array}{cc} \tau_{yz} & =\mu^{\prime }\frac{\partial w}{\partial y} \end{array}$$
  (A9) $$\begin{array}{cc} \tau_{zx} & =\mu \left(\frac{\partial w}{\partial x}+\frac{\partial u}{\partial z}\right) \end{array}$$
  where τ is the shear stress, *u* and *w* are the lateral and vertical components of the displacement vector, μ and $\mu^{\prime }$ are shear moduli, and $C_{1}$ and $C_{2}$ are constants [17]. This system of partial differential equations can be efficiently solved by finite element methods.

The following is a brief description of various VT models:

- **Bio-mechanical models**:
  Bio-mechanical models simulate the geometry and articulatory movements of the vocal tract using displacement-based finite element methods and take into account the continuous tissue deformation and variation of the physiological, bio-mechanical, and visco-elastic properties of muscles [27]. They are more scalable and accurate, and allow for the modeling of more fine-grained control over muscular forces, articulator positions, and movements.

- **Statistical models**:
  These model the vocal tract as statistical factors or components. For instance, factor analysis describes the vocal tract profile as a sum of articulatory components and analyzes the relationship between individual, or a combination of, components and vocal tract parameters [25].

- **Geometric models**:
  These attempt to depict the shape and geometric configurations of the vocal tract. They specify articulatory state with vocal tract parameters that define the position and shape of tongue, lips, jaw, larynx, etc. [26]. However, such models are not scalable because they do not account for the continuous variations of the anatomy and articulatory state, require clinical measurements such as from magnetic resonance imaging, and are not amendable to coupling with vocal fold models.

## Appendix B

### Appendix B.1. Modeling Wave Propagation in the Vocal Tract

The vocal tract can be viewed as a compact, orientable, differentiable manifold *M* embedded in $R^{3}$. Its boundary ∂M includes the wall of the vocal tract. Consider the tangent bundle TM. Denote the set of all vector fields on TM as Γ(TM), which is a $C^{\infty }\left(M\right)$-module [43]. A vector field is a smooth section on TM,Γ(TM)∋X:M→TM. It associates each point p∈M with a tangent vector $\bar{v}\left(p\right):={\left.X\right|}_{p}:C^{\infty }\left(M\right)\tilde{\to }R$[43]. Let γ(t):R⊇I→M be a maximal integral curve [43] through *p* at $t_{0}$, which is a solution to:

(A10) $$\begin{array}{cc} \gamma^{\prime }\left(t\right) & =X(\gamma (t))\\ \gamma \left(t_{0}\right) & =p \end{array}$$

The curve γ(t) is a one-parameter group. When acting on the Lie group *M*, it gives the flow $\Phi :R\times M\to M.\Phi_{t}\left(p\right)=\gamma \left(t\right)$. The particle velocity at p is given by $v\left(p,t\right):=\gamma^{\prime }\left(t\right)=$$\bar{v}\left(p\right)\circ \gamma \left(t\right)$

The planar motion of the pressure wave in the vocal tract is governed by the equations [36]:

(A11) $$\begin{array}{c} \frac{1}{\rho c^{2}}\frac{\partial \hat{p}}{\partial t}+\mathrm{div}\;v=0 \end{array}$$

(A12) $$\begin{array}{c} \rho \frac{\partial v}{\partial t}+\mathrm{grad}\;\hat{p}=0 \end{array}$$

where $\hat{p}\left(p,t\right)$ is the acoustic pressure, div is the divergence operator, grad is the gradient operator, ρ is the ambient air density, and *c* is the speed of sound. Equation (A11) describes the conservation of mass, and (A12) describes the conservation of momentum [36].

For notational convenience, we use cylindrical coordinates p=(r,θ,x), where the *x* direction aligns with the central axis of vocal tract. Denote the inner surface of vocal tract as Σ, and the shape function of inner surface as r=R(θ,x). Then the cross-sectional area of the vocal tract is:

(A13) $$A\left(x\right)=\int_{0}^{2\pi }d\theta \int_{0}^{R(\theta ,x)}rdr$$

The average acoustic pressure is:

(A14) $$p\left(x,t\right)=\frac{1}{A(x)}\int_{0}^{2\pi }d\theta \int_{0}^{R(\theta ,x)}\hat{p}rdr$$

and the volume velocity is:

(A15) $$u\left(x,t\right)=\int_{0}^{2\pi }d\theta \int_{0}^{R(\theta ,x)}v_{x}rdr$$

where $v_{x}$ is the *x* component of v. Integrating (A11) over the volume of vocal tract bounded by cross sections at $x_{0}$ and *x* gives:

(A16) $$\begin{array}{cc} 0 & =\int_{M}\frac{1}{\rho c^{2}}\frac{\partial \hat{p}}{\partial t}+\mathrm{div}v \end{array}$$

(A17) $$\begin{array}{cc} \; & \;\;\;\;\;\;\;\;=\int_{x_{0}}^{x}\left[\int_{0}^{2\pi }d\theta \int_{0}^{R}\frac{1}{\rho c^{2}}\frac{\partial \hat{p}}{\partial t}rdr\right]dx^{\prime }+\int_{M}\mathrm{div}v \end{array}$$

(A18) $$\begin{array}{cc} \; & \;\;\;\;\;\;\;\;\;\;\;\;\;\;=\frac{1}{\rho c^{2}}\int_{x_{0}}^{x}A\left(x^{\prime }\right)\frac{\partial p\left(x^{\prime },t\right)}{\partial t}dx^{\prime }+\underset{\Sigma }{\;\int \int \;}n_{v}d\sigma +u\left(x,t\right)-u\left(x_{0},t\right) \end{array}$$

where we substitute Equations (A17) and (A18) into (A14) and (A15), and apply Stokes’ theorem [28,36]; $n_{v}$ is the component of v normal and outward to the inner surface Σ.

The element of area dσ is given by [28,36]:

(A19) dσ=S(θ,x)dθdx

where Sdθdx is a top 2-form on Σ [43]. Substituting (A19) into (A18) and differentiating w.r.t. *x* yields:

(A20) $$\frac{A(x)}{\rho c^{2}}\frac{\partial p}{\partial t}+\frac{\partial u}{\partial x}+\int_{0}^{2\pi }n_{v}\left(\theta ,x,t\right)S\left(\theta ,x\right)d\theta =0$$

Following similar steps, integrating the *x* component of (A12) over the cross section at *x* yields:

(A21) $$\rho \frac{\partial u}{\partial t}+A\left(x\right)\frac{\partial p}{\partial x}+\int_{0}^{2\pi }\left(p\left(x,t\right)-p_{w}\left(\theta ,x,t\right)\right)\frac{\partial }{\partial x}\left(\frac{1}{2}R^{2}\right)d\theta =0$$

where $p_{w}$ is the pressure acting on the wall of the vocal tract.

### Appendix B.2. The Integrated Vocal Tract Model

To simplify our problem, we combine the wave Equations (A20) and (A21) into a single vocal tract model. Differentiating (A20) w.r.t *x* and (A21) w.r.t. *t*, and canceling out the pressure term gives:

$$\begin{array}{cc} \frac{\partial^{2}u}{\partial t^{2}}= & c^{2}\frac{\partial^{2}u}{\partial x^{2}}+\frac{1}{\rho }\frac{\partial A}{\partial x}\frac{\partial p}{\partial t}-\frac{1}{\rho }\partial_{t}\int_{0}^{2\pi }\left(p\left(x,t\right)-p_{w}\left(\theta ,x,t\right)\right)\frac{\partial }{\partial x}\left(\frac{1}{2}R^{2}\right)d\theta \end{array}$$

(A22) $$\begin{array}{cc} \; & +c^{2}\partial_{x}\int_{0}^{2\pi }n_{v}\left(\theta ,x,t\right)S\left(\theta ,x\right)d\theta \end{array}$$

(A23) $$\begin{array}{cc} = & c^{2}\frac{\partial^{2}u}{\partial x^{2}}+f\left(x,t\right) \end{array}$$

where the vocal tract profile is absorbed into a single term f(x,t). This represents the characteristics of the vocal tract, i.e., the effect of the nonuniform yielding wall on the acoustic flow dynamics, which needs to be estimated by our algorithm.

## Appendix C

In this appendix, we derive the ADLES algorithm to estimate VFO model parameters from a given glottal excitation signal.

Let $u_{g}\left(t\right)$ be the actual air volume velocity at the glottis. We can can also derive $u_{0}\left(t\right)$ from the displacement of the vocal folds as

(A24) $$u_{0}\left(t\right)=\tilde{c}d\left(2\xi_{0}+\xi_{l}\left(t\right)+\xi_{r}\left(t\right)\right)$$

where $\xi_{0}$ is the half glottal width at rest, *d* is the length of vocal fold, and $\tilde{c}$ is the air particle velocity at the midpoint of the vocal fold.

We assume the movements of the vocal folds to follow the dynamics specified by Equation (3) in Section 3.1. Our objective is then to estimate the parameters of the model to minimize the difference:

(A25) $$\begin{array}{cc} \; & \min\int_{0}^{T}{\left(u_{0}\left(t\right)-u_{g}\left(t\right)\right)}^{2}dt\Leftrightarrow \end{array}$$

(A26) $$\begin{array}{cc} \; & \min\int_{0}^{T}{\left(\tilde{c}d\left(2\xi_{0}+\xi_{l}\left(t\right)+\xi_{r}\left(t\right)\right)-u_{g}\left(t\right)\right)}^{2}dt \end{array}$$

such that the movements of the vocal folds follow the VFO model:

(A27) $$\begin{array}{cc} \; & {\ddot{\xi }}_{r}+\beta \left(1+\xi_{r}^{2}\right){\dot{\xi }}_{r}+\xi_{r}-\frac{\Delta }{2}\xi_{r}=\alpha \left({\dot{\xi }}_{r}+{\dot{\xi }}_{l}\right) \end{array}$$

(A28) $$\begin{array}{cc} \; & {\ddot{\xi }}_{l}+\beta \left(1+\xi_{l}^{2}\right){\dot{\xi }}_{l}+\xi_{l}+\frac{\Delta }{2}\xi_{l}=\alpha \left({\dot{\xi }}_{r}+{\dot{\xi }}_{l}\right) \end{array}$$

(A29) $$\begin{array}{cc} \; & \xi_{r}\left(0\right)=C_{r} \end{array}$$

(A30) $$\begin{array}{cc} \; & \xi_{l}\left(0\right)=C_{l} \end{array}$$

(A31) $$\begin{array}{cc} \; & {\dot{\xi }}_{r}\left(0\right)=0 \end{array}$$

(A32) $$\begin{array}{cc} \; & {\dot{\xi }}_{l}\left(0\right)=0 \end{array}$$

where $C_{r}$ and $C_{l}$ are constants representing the quiescent positions of the vocal folds.

### The Adjoint Least Squares (ADLES) Solution

To solve the functional least squares in (A26), we require the gradients of (A26) w.r.t. the model parameters α, β and Δ. Subsequently, we can adopt any gradient-based (local or global) method to obtain the solution.

Considering the residual

(A33) $$R=\tilde{c}d\left(2\xi_{0}+\xi_{l}\left(t\right)+\xi_{r}\left(t\right)\right)-u_{g}\left(t\right)$$

We have

(A34) $$f\left(\xi_{l},\xi_{r};\vartheta \right)=R^{2}$$

and

(A35) $$F\left(\xi_{l},\xi_{r};\vartheta \right)=\int_{0}^{T}f\left(\xi_{l},\xi_{r};\vartheta \right)dt$$

where ϑ=[α,β,Δ].

Incorporating the constraints into the objective using Lagrangian multipliers, we define the Lagrangian:

(A36) $$\begin{array}{cc} \; & L(\vartheta )=\\ \; & \int_{0}^{T}[f+\lambda \left({\ddot{\xi }}_{r}+\beta \left(1+\xi_{r}^{2}\right){\dot{\xi }}_{r}+\xi_{r}-\frac{\Delta }{2}\xi_{r}-\alpha \left({\dot{\xi }}_{r}+{\dot{\xi }}_{l}\right)\right)\\ \; & +\eta \left({\ddot{\xi }}_{l}+\beta \left(1+\xi_{l}^{2}\right){\dot{\xi }}_{l}+\xi_{l}+\frac{\Delta }{2}\xi_{l}-\alpha \left({\dot{\xi }}_{r}+{\dot{\xi }}_{l}\right)\right)]dt\\ \; & +\mu_{l}\left(\xi_{l}\left(0\right)-C_{l}\right)+\mu_{r}\left(\xi_{r}\left(0\right)-C_{r}\right)+\nu_{l}{\dot{\xi }}_{l}\left(0\right)+\nu_{r}{\dot{\xi }}_{r}\left(0\right) \end{array}$$

where λ, η, μ and ν are Lagrangian multipliers. Taking the derivative of the Lagrangian w.r.t. the model parameter α yields:

(A37) $$\begin{array}{cc} L_{\alpha } & =\int_{0}^{T}[2\tilde{c}dR\left(\partial_{\alpha }\xi_{l}+\partial_{\alpha }\xi_{r}\right)\\ \; & +\lambda (\partial_{\alpha }{\ddot{\xi }}_{r}+2\beta {\dot{\xi }}_{r}\xi_{r}\partial_{\alpha }\xi_{r}+\beta \left(1+\xi_{r}^{2}\right)\partial_{\alpha }{\dot{\xi }}_{r}\\ \; & +\partial_{\alpha }\xi_{r}-\frac{\Delta }{2}\partial_{\alpha }\xi_{r}-\alpha \left(\partial_{\alpha }{\dot{\xi }}_{r}+\partial_{\alpha }{\dot{\xi }}_{l}\right)-\left({\dot{\xi }}_{r}+{\dot{\xi }}_{l}\right))\\ \; & +\eta (\partial_{\alpha }{\ddot{\xi }}_{l}+2\beta {\dot{\xi }}_{l}\xi_{l}\partial_{\alpha }\xi_{l}+\beta \left(1+\xi_{l}^{2}\right)\partial_{\alpha }{\dot{\xi }}_{l}\\ \; & +\partial_{\alpha }\xi_{l}+\frac{\Delta }{2}\partial_{\alpha }\xi_{l}-\alpha \left(\partial_{\alpha }{\dot{\xi }}_{r}+\partial_{\alpha }{\dot{\xi }}_{l}\right)-\left({\dot{\xi }}_{r}+{\dot{\xi }}_{l}\right))]dt\\ \; & +\mu_{l}\partial_{\alpha }\xi_{l}\left(0\right)+\mu_{r}\partial_{\alpha }\xi_{r}\left(0\right)+\nu_{l}\partial_{\alpha }{\dot{\xi }}_{l}\left(0\right)+\nu_{r}\partial_{\alpha }{\dot{\xi }}_{r}\left(0\right) \end{array}$$

Integrating the term $\lambda \partial_{\alpha }{\ddot{\xi }}_{r}$ twice by parts yields:

(A38) $$\int_{0}^{T}\lambda \partial_{\alpha }{\ddot{\xi }}_{r}dt=\int_{0}^{T}\partial_{\alpha }\xi_{r}\ddot{\lambda }dt-{\left.\partial_{\alpha }\xi_{r}\dot{\lambda }\right|}_{0}^{T}+{\left.\partial_{\alpha }{\dot{\xi }}_{r}\lambda \right|}_{0}^{T}$$

Applying the same to $\eta \partial_{\alpha }{\ddot{\xi }}_{l}$, substituting into (A37) and simplifying the final expression we obtain:

(A39) $$\begin{array}{cc} L_{\alpha } & =\int_{0}^{T}[\left(\ddot{\lambda }+\left(2\beta \xi_{r}{\dot{\xi }}_{r}+1-\frac{\Delta }{2}\right)\lambda +2\tilde{c}dR\right)\partial_{\alpha }\xi_{r}\\ \; & +\left(\ddot{\eta }+\left(2\beta \xi_{l}{\dot{\xi }}_{l}+1+\frac{\Delta }{2}\right)\lambda +2\tilde{c}dR\right)\partial_{\alpha }\xi_{l}\\ \; & +\left(\beta \left(1+\xi_{r}^{2}\right)\lambda -\alpha \left(\lambda +\eta \right)\right)\partial_{\alpha }{\dot{\xi }}_{r}\\ \; & +\left(\left(\beta \left(1+\xi_{l}^{2}\right)\eta -\alpha \left(\lambda +\eta \right)\right)\partial_{\alpha }{\dot{\xi }}_{l}\right.\\ \; & -\left({\dot{\xi }}_{r}+{\dot{\xi }}_{l}\right)\left(\lambda +\eta \right)]dt\\ \; & +\left(\mu_{r}+\dot{\lambda }\right)\partial_{\alpha }\xi_{r}\left(0\right)-\dot{\lambda }\partial_{\alpha }\xi_{r}\left(T\right)\\ \; & +\left(\nu_{r}-\lambda \right)\partial_{\alpha }{\dot{\xi }}_{r}\left(0\right)+\lambda \partial_{\alpha }{\dot{\xi }}_{r}\left(T\right)\\ \; & +\left(\mu_{l}+\dot{\eta }\right)\partial_{\alpha }\xi_{l}\left(0\right)-\dot{\eta }\partial_{\alpha }\xi_{l}\left(T\right)\\ \; & +\left(\nu_{l}-\eta \right)\partial_{\alpha }{\dot{\xi }}_{l}\left(0\right)+\eta \partial_{\alpha }{\dot{\xi }}_{l}\left(T\right) \end{array}$$

Since the partial derivative of the model output ξ w.r.t. the model parameter α is difficult to compute, we eliminate the related terms by setting

For 0<t<T:

(A40) $$\begin{array}{cc} \; & \ddot{\lambda }+\left(2\beta \xi_{r}{\dot{\xi }}_{r}+1-\frac{\Delta }{2}\right)\lambda +2\tilde{c}dR=0 \end{array}$$

(A41) $$\begin{array}{cc} \; & \ddot{\eta }+\left(2\beta \xi_{l}{\dot{\xi }}_{l}+1+\frac{\Delta }{2}\right)\eta +2\tilde{c}dR=0 \end{array}$$

(A42) $$\begin{array}{cc} \; & \beta \left(1+\xi_{r}^{2}\right)\lambda -\alpha \left(\lambda +\eta \right)=0 \end{array}$$

(A43) $$\begin{array}{cc} \; & \beta \left(1+\xi_{l}^{2}\right)\eta -\alpha \left(\lambda +\eta \right)=0 \end{array}$$

with initial conditions at t=T:

(A44) $$\begin{array}{cc} \; & \lambda (T)=0 \end{array}$$

(A45) $$\begin{array}{cc} \; & \dot{\lambda }\left(T\right)=0 \end{array}$$

(A46) $$\begin{array}{cc} \; & \eta (T)=0 \end{array}$$

(A47) $$\begin{array}{cc} \; & \dot{\eta }\left(T\right)=0 \end{array}$$

As a result, we obtain the derivative of *F* w.r.t. α as:

(A48) $$\begin{array}{c} F_{\alpha }=\int_{0}^{T}-\left({\dot{\xi }}_{r}+{\dot{\xi }}_{l}\right)\left(\lambda +\eta \right)dt \end{array}$$

The derivatives of *F* w.r.t. β and Δ are similarly obtained as:

(A49) $$F_{\beta }=\int_{0}^{T}\left(\left(1+\xi_{r}^{2}\right){\dot{\xi }}_{r}\lambda +\left(1+\xi_{l}^{2}\right){\dot{\xi }}_{l}\eta \right)dt$$

(A50) $$F_{\Delta }=\int_{0}^{T}\frac{1}{2}\left(\xi_{l}\eta -\xi_{r}\lambda \right)dt$$

Having calculated the gradients of *F* w.r.t. the model parameters, we can now apply the gradient-descent rule to optimize our objective (A26):

(A51) $$\begin{array}{cc} \alpha^{k+1} & =\alpha^{k}-\tau^{\alpha }F_{\alpha }\\ \beta^{k+1} & =\beta^{k}-\tau^{\beta }F_{\beta }\\ \Delta^{k+1} & =\Delta^{k}-\tau^{\Delta }F_{\Delta } \end{array}$$

where $\tau^{\cdot }$ is the step-size.

The overall algorithm is summarized as follows:

1. Integrate (A27) and (A28) with initial conditions (A29)–(A32) from 0 to *T*, obtaining $\xi_{r}$, $\xi_{l}$, ${\dot{\xi }}_{r}$ and ${\dot{\xi }}_{l}$.
2. Integrate (A40)–(A43) with the initial conditions (A44)–(A47) from *T* to 0, obtaining λ, $\dot{\lambda }$, η and $\dot{\eta }$.
3. Update α, β and Δ with (A51).

## Appendix D

### Appendix D.1. The Combined Vocal Folds-Tract Model: Formulation of the Inverse Problem

In this appendix we formulate the problem of estimating the parameters of the combined vocal fold-tract model from speech measurements. Let Ω×T be the domain of volume velocity *u*, where Ω is the spatial domain, and T is the time domain. In the one-dimensional case, Ω=[0,L] where *L* is the length of vocal tract, and T=[0,T]. Given measured acoustic pressure $p_{m}\left(t\right)$ at the lip, the corresponding volume velocity is given by [34]:

(A52) $$u_{m}\left(t\right)=\frac{A(L)}{\rho c}p_{m}\left(t\right)$$

where A(L) is the opening area at the lip, *c* is the speed of sound, and ρ is the ambient air density. We denote $u_{0}\left(t\right)u\left(0,t\right)$, $u_{L}\left(t\right)u\left(L,t\right)$. The glottal flow $u_{0}\left(t\right)$ can be derived from the vocal folds displacement model (3) by:

(A53) $$u_{0}\left(t\right)=\tilde{c}d\left(2\xi_{0}+\xi_{l}\left(t\right)+\xi_{r}\left(t\right)\right)$$

where $\xi_{0}$ is the half glottal width at rest, *d* is the length of the vocal fold, and $\tilde{c}$ is the air particle velocity at the midpoint of the vocal fold (see Figure 3).

Let H be the nonlinear operator representing acoustic wave propagation from the glottis to the lip. We have the forward propagation process:

(A54) $$\begin{array}{cc} H:L^{2}\left(\Omega \times T\right)\times L^{2}\left(\Gamma \times T\right) & \to L^{2}\left(\Gamma \times T\right)\\ \left(f,u_{0}\right) & \mapsto u_{L} \end{array}$$

where *f* is the vocal tract profile in (A22), and Γ=∂Ω is the boundary.

We can split Γ into two parts $\Gamma =\Gamma_{0}⋃\Gamma_{1}$, $\Gamma_{0}⋂\Gamma_{L}=\emptyset$ corresponding to x=0 and x=L. However, we neglect the difference to ease our derivation. Note that in the one-dimensional case u(t) and $u_{L}\left(t\right)$ are only functions of *t*. However, more generally, they are functions of both *x* on the boundary Γ and *t*. We now define two nonlinear operators as:

(A55) $$\begin{array}{cc} H_{f}:L^{2}\left(\Gamma \times T\right) & \to L^{2}\left(\Gamma \times T\right)\\ u_{0} & \mapsto u_{L} \end{array}$$

and

(A56) $$\begin{array}{cc} F:=H_{u_{0}}:L^{2}\left(\Omega \times T\right) & \to L^{2}\left(\Gamma \times T\right)\\ f & \mapsto u_{L} \end{array}$$

Note that both $H_{f}$ and F are bounded. Our objective is to (estimate model parameters α, β and Δ such that they) minimize the difference between the measured volume velocity $u_{m}$ and the predicted volume velocity $u_{L}$ near the lip subject to constraints:

(A57) $$\begin{array}{cc} \; & \min\int_{0}^{T}{\left(H_{f}\left(u_{0}\left(t\right)\right)-u_{m}\left(t\right)\right)}^{2}dt \end{array}$$

(A58) $$\begin{array}{cc} \Leftrightarrow \; & \min\int_{0}^{T}{\left(H_{f}\left(\tilde{c}d\left(2\xi_{0}+\xi_{l}\left(t\right)+\xi_{r}\left(t\right)\right)\right)-\frac{A(L)}{\rho c}p_{m}\left(t\right)\right)}^{2}dt \end{array}$$

(A59) $$\begin{array}{cc} \;\mathrm{subject}\;\mathrm{to}\;\; & {\ddot{\xi }}_{r}+\beta \left(1+\xi_{r}^{2}\right){\dot{\xi }}_{r}+\xi_{r}-\frac{\Delta }{2}\xi_{r}=\alpha \left({\dot{\xi }}_{r}+{\dot{\xi }}_{l}\right) \end{array}$$

(A60) $$\begin{array}{cc} \; & {\ddot{\xi }}_{l}+\beta \left(1+\xi_{l}^{2}\right){\dot{\xi }}_{l}+\xi_{l}+\frac{\Delta }{2}\xi_{l}=\alpha \left({\dot{\xi }}_{r}+{\dot{\xi }}_{l}\right) \end{array}$$

(A61) $$\begin{array}{cc} \; & \;\left(I.C.1\right)\;\xi_{r}\left(0\right)=C_{r} \end{array}$$

(A62) $$\begin{array}{cc} \; & \;\left(I.C.2\right)\;\xi_{l}\left(0\right)=C_{l} \end{array}$$

(A63) $$\begin{array}{cc} \; & \;\left(I.C.3\right)\;{\dot{\xi }}_{r}\left(0\right)=0 \end{array}$$

(A64) $$\begin{array}{cc} \; & \;\left(I.C.4\right)\;{\dot{\xi }}_{l}\left(0\right)=0 \end{array}$$

where (30) and (A60) represent the asymmetric vocal folds displacement model (3), I.C. stands for initial condition, and *C*s are constants.

### Appendix D.2. Solving the Inverse Problem for the Vocal Folds-Tract Model: The ADLES-VFT Algorithm

We solve the inverse problem for the combined vocal folds-tract model using the Forward-Backward method proposed below. We call this algorithm the Adjoint Least Squares—Vocal Folds-Tract (ADLES-VFT) parameter estimation algorithm.

In order to solve the parameter estimation problem represented by (A60)–(A64), first we need to estimate the vocal tract profile *f* in H and (A23). Specifically, we need to solve:

(A65) $$\begin{array}{cc} \; & \frac{\partial^{2}u\left(x,t\right)}{\partial t^{2}}=c^{2}\frac{\partial^{2}u\left(x,t\right)}{\partial x^{2}}+f\left(x,t\right)\\ \;\mathrm{subject}\;\mathrm{to}\;\; & \end{array}$$

(A66) $$\begin{array}{cc} \;\left(B.C.1\right)\;\; & u\left(0,t\right)=u_{g}\left(t\right) \end{array}$$

(A67) $$\begin{array}{cc} \;\left(B.C.2\right)\;\; & \;\;\;\frac{\partial u}{\partial n_{\Gamma }}=0 \end{array}$$

(A68) $$\begin{array}{cc} \;\left(I.C.1\right)\;\; & \;\;u(x,0)=0 \end{array}$$

(A69) $$\begin{array}{cc} \;\left(I.C.2\right)\;\; & \frac{\;\partial u(x,0)}{\partial t}=0 \end{array}$$

where B.C. stands for boundary condition, $u_{g}$ is the volume velocity at the glottis and $n_{\Gamma }$ is the outward unit normal to the boundary Γ. We now derive the solution to (A65)–(A69).

In order to estimate $f\in L^{2}\left(\Omega \times T\right)$, we take an iterative approach, i.e.,

(A70) $$f^{k+1}=f^{k}+\tau \delta f^{k}$$

where $\delta f^{k}\in L^{2}\left(\Omega \times T\right)$ is a small variation, and τ is a step size. A Taylor expansion of F (A56) at $f^{k}$ gives:

(A71) $$F\left(f^{k}+\delta f^{k}\right)=F\left(f^{k}\right)+F^{\prime }\left(f^{k}\right)\delta f^{k}+O\left({\left(\delta f^{k}\right)}^{2}\right)$$

where $F^{\prime }$ is the Fréchet derivative [44]. Omitting higher order terms, we obtain:

(A72) $$F^{\prime }\left(f^{k}\right)\delta f^{k}=F\left(f^{k}+\delta f^{k}\right)-F\left(f^{k}\right)$$

where $F^{\prime }\left(f\right)$ is a nonlinear operator

(A73) $$\begin{array}{ccc} F^{\prime }\left(f\right): & L^{2}\left(\Omega \times T\right) & \to L^{2}\left(\Gamma \times T\right)\\ \delta f & \mapsto \delta u_{L} \end{array}$$

Correspondingly, the adjoint operator [44,45,46] is:

(A74) $$\begin{array}{ccc} F^{\prime }{\left(f\right)}^{*}: & L^{2}\left(\Gamma \times T\right) & \to L^{2}\left(\Omega \times T\right)\\ \delta u_{L} & \mapsto \delta f \end{array}$$

We would like $F\left(f^{k}+\delta f^{k}\right)=u_{L}^{k}+\delta u_{L}^{k}\overset{k\to \infty }{\to }u_{m}$. This is equivalent to solving:

$$\begin{array}{cc} \; & \min{∥\delta f^{k}∥}_{2}^{2}\\ \;\mathrm{subject}\;\mathrm{to}\; & F^{\prime }\left(f^{k}\right)\delta f^{k}=u_{m}-F\left(f^{k}\right) \end{array}$$

It is simple to obtain the solution to (A75):

(A75) $$\delta f^{k}=-F^{\prime }{\left(f^{k}\right)}^{*}{\left[F^{\prime }\left(f^{k}\right)F^{\prime }{\left(f^{k}\right)}^{*}\right]}^{-1}\left(F\left(f^{k}\right)-u_{m}\right)$$

where $F^{\prime }{\left(f^{k}\right)}^{*}$ is the adjoint operator. It is difficult to compute $F^{\prime }\left(f^{k}\right)F^{\prime }{\left(f^{k}\right)}^{*}$. We use its property of positive-definiteness to approximate it by γI where I is the identity matrix.

We denote the estimation residual as:

(A76) $$r^{k}:=u_{m}-F\left(f^{k}\right)$$

We have

(A77) $$\delta f^{k}=\frac{1}{\gamma }F^{\prime }{\left(f^{k}\right)}^{*}r^{k}$$

Now consider the wave Equation (A65). Let u+δu be a solution with variation f+δf. Substitution into (A65) yields:

(A78) $$\frac{\partial^{2}\left(u+\delta u\right)}{\partial t^{2}}=c^{2}\frac{\partial^{2}\left(u+\delta u\right)}{\partial x^{2}}+f+\delta f$$

Subtracting (A65) yields:

(A79) $$\begin{array}{cc} \; & \frac{\partial^{2}\delta u}{\partial t^{2}}=c^{2}\frac{\partial^{2}\delta u}{\partial x^{2}}+\delta f\\ \;\mathrm{subject}\;\mathrm{to}\; & \end{array}$$

(A80) $$\begin{array}{cc} \;\left(B.C.1\right)\; & \frac{\partial \delta u}{\partial n_{\Gamma }}=0 \end{array}$$

(A81) $$\begin{array}{cc} \;\left(I.C.1\right)\; & \delta u(x,0)=0 \end{array}$$

(A82) $$\begin{array}{cc} \;\left(I.C.2\right)\; & \frac{\partial \delta u(x,0)}{\partial t}=0 \end{array}$$

Next, we use a time reversal technique [36] and backpropagate the difference (A76) into the vocal tract, which gives:

(A83) $$\begin{array}{cc} \; & \frac{\partial^{2}z}{\partial t^{2}}=c^{2}\frac{\partial^{2}z}{\partial x^{2}}+f\left(x,t\right)\\ \;\mathrm{subject}\;\mathrm{to}\;\; & \end{array}$$

(A84) $$\begin{array}{cc} \;\left(B.C.1\right)\;\; & \frac{\partial z}{\partial n_{\Gamma }}=r \end{array}$$

(A85) $$\begin{array}{cc} \;\left(I.C.1\right)\;\; & z\left(x,T\right)=0 \end{array}$$

(A86) $$\begin{array}{cc} \;\left(I.C.2\right)\;\; & \frac{\partial z\left(x,T\right)}{\partial t}=0 \end{array}$$

where *z* is the time reversal of *u*. It follows [47] that:

(A87) $$\begin{array}{cc} \; & {\langle \delta f,z\rangle }_{\Omega \times T}=\int_{0}^{T}\int_{\Omega }\delta fzdxdt \end{array}$$

(A88) $$\begin{array}{cc} \; & =\int_{0}^{T}\int_{\Omega }\left(\frac{\partial^{2}\delta u}{\partial t^{2}}-c^{2}\frac{\partial^{2}\delta u}{\partial x^{2}}\right)zdxdt \end{array}$$

(A89) $$\begin{array}{cc} \; & =\int_{0}^{T}\int_{\Omega }\left(\frac{\partial^{2}\delta u}{\partial t^{2}}-c^{2}\frac{\partial^{2}\delta u}{\partial x^{2}}\right)zdxdt-\int_{0}^{T}\int_{\Omega }\left(\frac{\partial^{2}z}{\partial t^{2}}-c^{2}\frac{\partial^{2}z}{\partial x^{2}}-f\right)\delta udxdt\\ \; & =\int_{0}^{T}\int_{\Omega }\left(\frac{\partial^{2}\delta u}{\partial t^{2}}z-\frac{\partial^{2}z}{\partial t^{2}}\delta u\right)dxdt-c^{2}\int_{0}^{T}\int_{\Omega }\left(\frac{\partial^{2}\delta u}{\partial x^{2}}z-\frac{\partial^{2}z}{\partial x^{2}}\delta u\right)dxdt \end{array}$$

(A90) $$\begin{array}{cc} \; & +\int_{0}^{T}\int_{\Omega }f\delta udxdt\\ \; & ={\left.\int_{\Omega }\left(\frac{\partial \delta u}{\partial t}z-\frac{\partial z}{\partial t}\delta u\right)\right|}_{0}^{T}dxdt-c^{2}\int_{0}^{T}\int_{\Omega }\left(\frac{\partial^{2}\delta u}{\partial x^{2}}z-\frac{\partial^{2}z}{\partial x^{2}}\delta u\right)dxdt \end{array}$$

(A91) $$\begin{array}{cc} \; & +\int_{0}^{T}\int_{\Omega }f\delta udxdt \end{array}$$

(A92) $$\begin{array}{cc} \; & =-c^{2}\int_{0}^{T}\int_{\Omega }\left(\frac{\partial^{2}\delta u}{\partial x^{2}}z-\frac{\partial^{2}z}{\partial x^{2}}\delta u\right)dxdt+\int_{0}^{T}\int_{\Omega }f\delta udxdt \end{array}$$

(A93) $$\begin{array}{cc} \; & =-c^{2}\int_{0}^{T}\int_{\Omega }\left(zd\frac{\partial \delta u}{\partial x}-\delta ud\frac{\partial z}{\partial x}\right)dt+\int_{0}^{T}\int_{\Omega }f\delta udxdt\\ \; & =-c^{2}\int_{0}^{T}\left(\int_{\Gamma }z\frac{\partial \delta u}{\partial n_{\Gamma }}ds-\int_{\Omega }\frac{\partial \delta u}{\partial x}\frac{\partial z}{\partial x}dx-\int_{\Gamma }\delta u\frac{\partial z}{\partial n_{\Gamma }}ds+\int_{\Omega }\frac{\partial \delta u}{\partial x}\frac{\partial z}{\partial x}dx\right)dt \end{array}$$

(A94) $$\begin{array}{cc} \; & +\int_{0}^{T}\int_{\Omega }f\delta udxdt \end{array}$$

(A95) $$\begin{array}{cc} \; & =c^{2}\int_{0}^{T}\int_{\Gamma }\delta u\frac{\partial z}{\partial n_{\Gamma }}dsdt+\int_{0}^{T}\int_{\Omega }f\delta udxdt \end{array}$$

(A96) $$\begin{array}{cc} \; & =c^{2}\int_{0}^{T}\int_{\Gamma }\delta urdsdt+\int_{0}^{T}\int_{\Omega }f\delta udxdt \end{array}$$

(A97) $$\begin{array}{cc} \; & =c^{2}\int_{0}^{T}\int_{\Gamma }F^{\prime }\left(f\right)\delta frdsdt+\int_{0}^{T}\int_{\Omega }f\delta udxdt \end{array}$$

(A98) $$\begin{array}{cc} \; & =c^{2}\int_{0}^{T}\int_{\Omega }\delta fF^{\prime }{\left(f\right)}^{*}rdxdt+\int_{0}^{T}\int_{\Omega }f\delta udxdt \end{array}$$

(A99) $$\begin{array}{cc} \; & =c^{2}\int_{0}^{T}\int_{\Omega }\delta fF^{\prime }{\left(f\right)}^{*}rdxdt-\int_{0}^{T}\int_{\Omega }f\delta udxdt \end{array}$$

(A100) $$\begin{array}{cc} \; & =c^{2}\int_{0}^{T}\int_{\Omega }\delta f\left(F^{\prime }{\left(f\right)}^{*}r-u\right)dxdt \end{array}$$

where we substitute from (A87)–(A89) into (A79) and (A83); from (A89)–(A92) we apply initial conditions (A81), (A82), (A85) and (A86); from (A92)–(A94) we integrate by parts; from (A94)–(A95) we apply boundary condition (A80); from (A95)–(A96) we use boundary condition (A84); from (A96)–(A97) we use definition (A73); from (A97)–(A98) we use definition (A74) and the duality property

(A101) $${\langle F^{\prime }\left(f\right)\delta f,r\rangle }_{\Gamma \times T}={\langle \delta f,F^{\prime }{\left(f\right)}^{*}r\rangle }_{\Omega \times T}$$

from (A98)–(A99), we assume that the second-order variation is small, i.e.,

(A102) 〈f+δf,u+δu〉=〈f,u〉+〈f,δu〉+〈δf,u〉+〈δf,δu〉≈〈f,u〉

(or δ(fu)=δ(f)u+fδ(u)≈0). By the arbitrariness of δf, it follows that:

(A103) $$z=c^{2}\left(F^{\prime }{\left(f\right)}^{*}r-u\right)$$

and hence

(A104) $$F^{\prime }{\left(f\right)}^{*}r=\frac{z}{c^{2}}+u$$

Substitution into (A77) and (A70) yields:

(A105) $$f^{k+1}=f^{k}+\iota \left(\frac{z^{k}}{c^{2}}+u^{k}\right)$$

where ι=τ/γ.

Hence, we obtain an iterative forward-backward approach for solving the vocal tract profile *f*.

#### Appendix D.2.1. Estimating model parameters via the adjoint least squares method

Now, we derive solution to the parameter estimation problem (A60)–(A64) using the adjoint least squares method of Appendix C as follows.

Denote the estimation error as:

(A106) $$f\left(\xi_{l},\xi_{r};\vartheta \right)={\left(H_{f}\left(\tilde{c}d\left(2\xi_{0}+\xi_{l}\left(t\right)+\xi_{r}\left(t\right)\right)\right)-\frac{A(L)}{\rho c}p_{m}\left(t\right)\right)}^{2}$$

and

(A107) $$F\left(\xi_{l},\xi_{r};\vartheta \right)=\int_{0}^{T}f\left(\xi_{l},\xi_{r};\vartheta \right)dt$$

where ϑ=[α,β,Δ] are the parameters of the vocal folds model (3). We would like to obtain update rules for the model parameters α, β, and Δ, i.e.,

(A108) $$\begin{array}{cc} \alpha^{k+1} & =\alpha^{k}-\tau^{\alpha }F_{\alpha^{k}} \end{array}$$

(A109) $$\beta^{k+1}=\beta^{k}-\tau^{\beta }F_{\beta^{k}}$$

(A110) $$\Delta^{k+1}=\Delta^{k}-\tau^{\Delta }F_{\Delta^{k}}$$

where the the partial derivatives $F_{\cdot }\partial_{\cdot }F\equiv \frac{\partial F}{\partial \cdot }$ and $\tau^{\cdot }$ is the step size. We now define the Lagrangian:

(A111) $$\begin{array}{cc} L(\vartheta )= & \int_{0}^{T}\left[f+\lambda \left({\ddot{\xi }}_{r}+\beta \left(1+\xi_{r}^{2}\right){\dot{\xi }}_{r}+\xi_{r}-\frac{\Delta }{2}\xi_{r}-\alpha \left({\dot{\xi }}_{r}+{\dot{\xi }}_{l}\right)\right)\right.\\ \; & \left.+\eta \left({\ddot{\xi }}_{l}+\beta \left(1+\xi_{l}^{2}\right){\dot{\xi }}_{l}+\xi_{l}+\frac{\Delta }{2}\xi_{l}-\alpha \left({\dot{\xi }}_{r}+{\dot{\xi }}_{l}\right)\right)\right]dt\\ \; & +\mu_{l}\left(\xi_{l}\left(0\right)-C_{l}\right)+\mu_{r}\left(\xi_{r}\left(0\right)-C_{r}\right)+\nu_{l}{\dot{\xi }}_{l}\left(0\right)+\nu_{r}{\dot{\xi }}_{r}\left(0\right) \end{array}$$

where λ, η, μ and ν are multipliers. Taking the derivative of the Lagrangian w.r.t. the model parameter α yields

(A112) $$\begin{array}{cc} \; & L_{\alpha }=\int_{0}^{T}[{\left.2\tilde{c}dH_{f}^{\prime }\right|}_{u_{0}}\left(\partial_{\alpha }\xi_{l}+\partial_{\alpha }\xi_{r}\right)\\ \; & +\lambda (\partial_{\alpha }{\ddot{\xi }}_{r}+2\beta {\dot{\xi }}_{r}\xi_{r}\partial_{\alpha }\xi_{r}+\beta \left(1+\xi_{r}^{2}\right)\partial_{\alpha }{\dot{\xi }}_{r}+\partial_{\alpha }\xi_{r}\\ \; & -\frac{\Delta }{2}\partial_{\alpha }\xi_{r}-\alpha \left(\partial_{\alpha }{\dot{\xi }}_{r}+\partial_{\alpha }{\dot{\xi }}_{l}\right)-\left({\dot{\xi }}_{r}+{\dot{\xi }}_{l}\right))\\ \; & +\eta (\partial_{\alpha }{\ddot{\xi }}_{l}+2\beta {\dot{\xi }}_{l}\xi_{l}\partial_{\alpha }\xi_{l}+\beta \left(1+\xi_{l}^{2}\right)\partial_{\alpha }{\dot{\xi }}_{l}+\partial_{\alpha }\xi_{l}\\ \; & +\frac{\Delta }{2}\partial_{\alpha }\xi_{l}-\alpha \left(\partial_{\alpha }{\dot{\xi }}_{r}+\partial_{\alpha }{\dot{\xi }}_{l}\right)-\left({\dot{\xi }}_{r}+{\dot{\xi }}_{l}\right))]dt\\ \; & +\mu_{l}\partial_{\alpha }\xi_{l}\left(0\right)+\mu_{r}\partial_{\alpha }\xi_{r}\left(0\right)+\nu_{l}\partial_{\alpha }{\dot{\xi }}_{l}\left(0\right)+\nu_{r}\partial_{\alpha }{\dot{\xi }}_{r}\left(0\right) \end{array}$$

Integrating the term $\lambda \partial_{\alpha }{\ddot{\xi }}_{r}$ by parts twice gives:

(A113) $$\int_{0}^{T}\lambda \partial_{\alpha }{\ddot{\xi }}_{r}dt=\int_{0}^{T}\partial_{\alpha }\xi_{r}\ddot{\lambda }dt-{\left.\partial_{\alpha }\xi_{r}\dot{\lambda }\right|}_{0}^{T}+{\left.\partial_{\alpha }{\dot{\xi }}_{r}\lambda \right|}_{0}^{T}$$

Defining the estimation residual $R\;:=H_{f}\left(u_{0}\right)-\frac{A(L)}{\rho c}p_{m}\left(t\right)$, applying this to $\eta \partial_{\alpha }{\ddot{\xi }}_{l}$, and substituting into (A112), after simplification yields:

(A114) $$\begin{array}{cc} \; & L_{\alpha }=\int_{0}^{T}[\left(\ddot{\lambda }+\left(2\beta \xi_{r}{\dot{\xi }}_{r}+1-\frac{\Delta }{2}\right)\lambda +{\left.2\tilde{c}dRH_{f}^{\prime }\right|}_{u_{0}}\right)\partial_{\alpha }\xi_{r}\\ \; & +\left(\ddot{\eta }+\left(2\beta \xi_{l}{\dot{\xi }}_{l}+1+\frac{\Delta }{2}\right)\lambda +{\left.2\tilde{c}dRH_{f}^{\prime }\right|}_{u_{0}}\right)\partial_{\alpha }\xi_{l}\\ \; & +\left(\beta \left(1+\xi_{r}^{2}\right)\lambda -\alpha \left(\lambda +\eta \right)\right)\partial_{\alpha }{\dot{\xi }}_{r}+\left(\beta \left(1+\xi_{l}^{2}\right)\eta -\alpha \left(\lambda +\eta \right)\right)\partial_{\alpha }{\dot{\xi }}_{l}-\\ \; & \left({\dot{\xi }}_{r}+{\dot{\xi }}_{l}\right)\left(\lambda +\eta \right)]dt\\ \; & +\left(\mu_{r}+\dot{\lambda }\right)\partial_{\alpha }\xi_{r}\left(0\right)-\dot{\lambda }\partial_{\alpha }\xi_{r}\left(T\right)+\left(\nu_{r}-\lambda \right)\partial_{\alpha }{\dot{\xi }}_{r}\left(0\right)+\lambda \partial_{\alpha }{\dot{\xi }}_{r}\left(T\right)\\ \; & +\left(\mu_{l}+\dot{\eta }\right)\partial_{\alpha }\xi_{l}\left(0\right)-\dot{\eta }\partial_{\alpha }\xi_{l}\left(T\right)+\left(\nu_{l}-\eta \right)\partial_{\alpha }{\dot{\xi }}_{l}\left(0\right)+\eta \partial_{\alpha }{\dot{\xi }}_{l}\left(T\right) \end{array}$$

where the term $H_{f}^{{}^{\prime }}{|}_{u_{0}}\approx u_{L}/u_{0}$ by linearization. Since the partial derivatives of the displacement ξ w.r.t. the model parameter α are difficult to compute, we cancel out the related terms by setting:

For 0<t<T :

(A115) $$\ddot{\lambda }+\left(2\beta \xi_{r}{\dot{\xi }}_{r}+1-\frac{\Delta }{2}\right)\lambda +{\left.2\tilde{c}dRH_{f}^{\prime }\right|}_{u_{0}}=0$$

(A116) $$\ddot{\eta }+\left(2\beta \xi_{l}{\dot{\xi }}_{l}+1+\frac{\Delta }{2}\right)\eta +{\left.2\tilde{c}dRH_{f}^{\prime }\right|}_{u_{0}}=0$$

(A117) $$\beta \left(1+\xi_{r}^{2}\right)\lambda -\alpha \left(\lambda +\eta \right)=0$$

(A118) $$\beta \left(1+\xi_{l}^{2}\right)\eta -\alpha \left(\lambda +\eta \right)=0$$

with initial conditions:

At t=T :

(A119) $$\lambda \left(T\right)=0$$

(A120) $$\dot{\lambda }\left(T\right)=0$$

(A121) $$\eta \left(T\right)=0$$

(A122) $$\dot{\eta }\left(T\right)=0$$

Assuming the boundary conditions on $\xi_{l}$ and $\xi_{r}$ to hold—an assumption we empirically find to be valid for $C_{r}=C_{l}=0$, we obtain the derivative of *F* w.r.t. α:

(A123) $$F_{\alpha }=\int_{0}^{T}-\left({\dot{\xi }}_{r}+{\dot{\xi }}_{l}\right)\left(\lambda +\eta \right)dt$$

Similarly, we obtain the derivatives of *F* w.r.t. β and Δ

(A124) $$F_{\beta }=\int_{0}^{T}\left(\left(1+\xi_{r}^{2}\right){\dot{\xi }}_{r}\lambda +\left(1+\xi_{l}^{2}\right){\dot{\xi }}_{l}\eta \right)dt$$

(A125) $$F_{\Delta }=\int_{0}^{T}\frac{1}{2}\left(\xi_{l}\eta -\xi_{r}\lambda \right)dt$$

#### Appendix D.2.2. The ADLES-VFT Algorithm Summarized

The algorithm for solving the parameter estimation problem (A60)–(A64) is outlined below.

1. Integrate (A59) and (A60) with initial conditions (A61)–(A64) from 0 to *T*, obtaining $\xi_{r}^{k}$, $\xi_{l}^{k}$, ${\dot{\xi }}_{r}^{k}$ and ${\dot{\xi }}_{l}^{k}$.
2. Solve the forward propagation model (A65)–(A69) for $u_{L}^{k}$, $H_{f}^{{}^{\prime }}{|}_{u_{0}^{k}}$.
3. Calculate the estimation difference $r^{k}$ using (A76).
4. Solve the backward propagation model (A83)–(A86) for $z^{k}$.
5. Update $f^{k}$ using (A105).
6. Integrate (A115)–(A118) with initial conditions (A119)–(A122) from *T* to 0, obtaining $\lambda^{k}$, ${\dot{\lambda }}^{k}$, $\eta^{k}$ and ${\dot{\eta }}^{k}$.
7. Update α, β and Δ with (A110).

## Appendix E

### Appendix E.1. Numerical Solution for Wave Propagation

In this appendix we describe a finite-element solution for time-dependent systems of PDEs examplified by the wave propagation equation:

(A126) $$\begin{array}{cc} \; & \frac{\partial^{2}u\left(x,t\right)}{\partial t^{2}}=c^{2}\frac{\partial^{2}u\left(x,t\right)}{\partial x^{2}}+f\left(x,t\right)\\ \;\mathrm{subject}\;\mathrm{to}\;\; & \end{array}$$

(A127) $$\begin{array}{cc} \;\left(B.C.1\right)\;\; & u\left(0,t\right)=u_{g}\left(t\right) \end{array}$$

(A128) $$\begin{array}{cc} \;\left(B.C.2\right)\;\; & \frac{\partial u}{\partial n_{\Gamma }}=0 \end{array}$$

(A129) $$\begin{array}{cc} \;\left(I.C.1\right)\;\; & u(x,0)=0 \end{array}$$

(A130) $$\begin{array}{cc} \;\left(I.C.2\right)\;\; & \frac{\partial u(x,0)}{\partial t}=0 \end{array}$$

and the reverse propagation equation:

(A131) $$\begin{array}{cc} \; & \frac{\partial^{2}z}{\partial t^{2}}=c^{2}\frac{\partial^{2}z}{\partial x^{2}}+f\left(x,t\right)\\ \;\mathrm{subject}\;\mathrm{to}\;\; & \end{array}$$

(A132) $$\begin{array}{cc} \;\left(B.C.1\right)\;\; & \frac{\partial z}{\partial n_{\Gamma }}=r \end{array}$$

(A133) $$\begin{array}{cc} \;\left(I.C.1\right)\;\; & z\left(x,T\right)=0 \end{array}$$

(A134) $$\begin{array}{cc} \;\left(I.C.2\right)\;\; & \frac{\partial z\left(x,T\right)}{\partial t}=0 \end{array}$$

where the solution must be defined for x∈(0,L) and t∈(0,T).

#### Appendix E.1.1. Variational Formulation

First, for the time-dependent system of PDEs, we discretize it along time *t* with the backward Euler method [48], yielding a sequence of differential equations. We split the time domain *T* into *N* uniform length intervals Δt. For time step *n*, 0≤n≤N−1, applying the backward Euler method to the left side of (A65) gives:

(A135) $${\left[D_{t}D_{t}^{-}u\right]}^{n}:={\left.D_{t}D_{t}^{-}\left(\frac{\partial^{2}u}{\partial t^{2}}\right)\right|}_{t=n\Delta t}=\frac{u^{n}-2u^{n-1}+u^{n-2}}{\Delta t^{2}}$$

where $D_{t}D_{t}^{-}$ is a finite difference operator w.r.t. time, and the superscript *n* indicates that the differencing is performed at time step *n* [48,49]. Substitution into (36) yields:

(A136) $${\left[D_{t}D_{t}^{-}u=c^{2}\frac{\partial^{2}u}{\partial x^{2}}+f\right]}^{n}$$

(A137) $$\Leftrightarrow \;u^{n}=\Delta t^{2}c^{2}\frac{\partial^{2}u^{n}}{\partial x^{2}}+\Delta t^{2}f^{n}+2u^{n-1}-u^{n-2}$$

Next, we define the residual at time step *n* as:

(A138) $$R^{n}=u^{n}-\Delta t^{2}c^{2}\frac{\partial^{2}u^{n}}{\partial x^{2}}+\Delta t^{2}f^{n}+2u^{n-1}-u^{n-2}$$

Applying Galerkin’s method [48,50] gives:

(A139) $${\langle R^{n},v\rangle }_{W^{k,2}}=0$$

where $v\in W^{k,2}$ ($W^{k,2}$ is the Sobolev space of functions with bounded $L_{2}$ norm and *k*-th order weak derivatives) is a qualified test function. Galerkin’s method orthogonally projects the residual to the function space $W^{k,2}$. Expanding (A139) yields:

(A140) $$\int_{\Omega }u^{n}vdx-\Delta t^{2}c^{2}\int_{\Omega }\frac{\partial^{2}u^{n}}{\partial x^{2}}vdx=\int_{\Omega }\left(\Delta t^{2}f^{n}+2u^{n-1}-u^{n-2}\right)vdx$$

Integration by parts for the second-order term in (A140) gives:

(A141) $$\int_{\Omega }\frac{\partial^{2}u^{n}}{\partial x^{2}}vdx=-\int_{\Omega }\frac{\partial u^{n}}{\partial x}\frac{\partial v}{\partial x}dx+\int_{\Gamma }\frac{\partial u^{n}}{\partial n_{\Gamma }}ds$$

where $n_{\Gamma }$ is the outward normal unit vector of the boundary Γ, and ds is the 1-form [43] on Γ. For problem (A126)–(A130), applying the boundary condition (A128) and substituting (A141) back into (A140) yields the variational problem:

(A142) $$\int_{\Omega }u^{n}vdx+\Delta t^{2}c^{2}\int_{\Omega }\frac{\partial u^{n}}{\partial x}\frac{\partial v}{\partial x}dx=\int_{\Omega }\left(\Delta t^{2}f^{n}+2u^{n-1}-u^{n-2}\right)vdx$$

For the reverse problem represented by (A131)–(A134), applying the boundary condition (A132) and substituting (A141) back into (A140) yields a similar variational problem:

(A143) $$\int_{\Omega }z^{n}wdx+\Delta t^{2}c^{2}\int_{\Omega }\frac{\partial z^{n}}{\partial x}\frac{\partial w}{\partial x}dx=\int_{\Omega }\left(\Delta t^{2}f^{n}+2z^{n-1}-z^{n-2}\right)wdx+\int_{\Gamma }r^{n}wds$$

We can split the variational problem (A142) into two parts:

(A144) $$a\left(u,v\right)=\int_{\Omega }uvdx+\Delta t^{2}c^{2}\int_{\Omega }\frac{\partial u}{\partial x}\frac{\partial v}{\partial x}dx$$

(A145) $$L\left(v\right)=\int_{\Omega }\left(\Delta t^{2}f^{n}+2u^{n-1}-u^{n-2}\right)vdx$$

where we have interchanged the unknown $u^{n}$ with *u*. (A144) is the bilinear form, and (A145) is the linear form [48].

Our original problem (A126)–(A130) and (A131)–(A134) then reduce to solving:

(A146) a(u,v)=L(v)

for each time step. By the Lax-Milgram Lemma [49], solving (A146) is equivalent to solving the functional minimization problem:

(A147) $$F\left(u\right)=\underset{v\in V}{\arg\min}\frac{1}{2}a\left(v,v\right)-L\left(v\right)$$

By the calculus of variations, and taking the variation of the functional gives (A146), hence the name *variational* form [48,49].

#### Appendix E.1.2. Finite Element Approximation

For each time step, we solve (A146) with finite element method. We discretize the domain Ω with a mesh of uniformly spaced triangular cells. We take the $P_{2}$ elements as the basis function space, which contains piece-wise, second-order Lagrange polynomials defined over a cell. Each basis function has a degree-of-freedom (DoF) of 6 over a two-dimensional cell [48,51].

Each element is associated with a coordinate map that transforms local coordinates to global coordinates and a DoF map that maps local DoF to global DoF [48,51]. Each cell is essentially a simplex and can be continuously transformed into the physical domain. We use the following two previously established points in this context, and proceed as explained thereafter.

1. **Existence of Unique Solution**: The solution to the variational problem (A146) exists and is unique [51].
2. **Approximation Error**: The Galerkin’s method gives the solution $u_{e}$ with error bounded by $O(h^{3}∥D^{2}u_{e}{∥}_{W^{3,2}})$, where *h* is the cell size and *D* is the bounded derivative operator [49,51].

Assume a solution $u=B+c^{j}\psi_{j}$ (using Einstein summation convention) with basis $\psi_{j}\in P_{2}$ and coefficients $c^{j}$. Here the $\psi_{j}$ are piece-wise second-order Lagrange polynomials [48]. Also, the mesh of cells, $P_{2}$ elements of basis functions, coordinate map, DoF map, and the assembly of the linear systems, are implemented and solved with the FEniCS software [52].

The function B(x) incorporates the boundary condition and, as an example, can take the form:

(A148) $$B\left(x\right)=u_{g}+\left(u_{m}-u_{g}\right)\frac{x^{p}}{L^{p}},\;p>0$$

We also project B(x) over the basis functions $P_{2}$ and express it as $B\left(x\right)=b^{j}\psi_{j}$. As a result, we obtain an unified expression $u=U^{j}\psi_{j}$ with $U^{j}$ incorporating $b^{j}$ and $c^{j}$. Similarly, we have $f^{n}=F_{n}^{j}\psi_{j}$, $u^{n-1}=U_{n-1}^{j}\psi_{j}$, $u^{n-2}=U_{n-2}^{j}\psi_{j}$. Set the test function as $v={\hat{\psi }}_{i}$ (where ${\hat{\psi }}_{i}$ is the $i^{\mathrm{th}}$ Lagrange polynomial basis in a test cell). Substitution into (A144) and (A145) yields:

(A149) $$\begin{array}{ll} a\left(u,v\right) & =\int_{\Omega }U^{j}\psi_{j}{\hat{\psi }}_{i}dx+\Delta t^{2}c^{2}\int_{\Omega }U^{j}\psi_{j}^{\prime }\psi_{i}^{\prime }dx\\ & =\left(\int_{\Omega }{\hat{\psi }}_{i}\psi_{j}dx+\Delta t^{2}c^{2}\int_{\Omega }\psi_{i}^{\prime }\psi_{j}^{\prime }dx\right)U^{j} \end{array}$$

(A150) $$\begin{array}{ll} L\left(v\right) & =\int_{\Omega }\left(\Delta t^{2}F_{n}^{j}\psi_{j}+2U_{n-1}^{j}\psi_{j}-U_{n-2}^{j}\psi_{j}\right){\hat{\psi }}_{i}dx\\ & =\Delta t^{2}\left(\int_{\Omega }{\hat{\psi }}_{i}\psi_{j}dx\right)F_{n}^{j}+2\left(\int_{\Omega }{\hat{\psi }}_{i}\psi_{j}dx\right)U_{n-1}^{j}-\left(\int_{\Omega }{\hat{\psi }}_{i}\psi_{j}dx\right)U_{n-2}^{j} \end{array}$$

where $\psi_{i}^{\prime }$ is the derivative of $\psi_{i}$ w.r.t. *x*.

Setting $M_{i,j}=\int_{\Omega }{\hat{\psi }}_{i}\psi_{j}dx$, $K_{i,j}=\int_{\Omega }\psi_{i}^{\prime }\psi_{j}^{\prime }dx$ and collecting (A149) and (A150) into matrix-vector form, we obtain:

(A151) AU=b

where $A=M+\Delta t^{2}c^{2}K$, and $b=\Delta t^{2}MF^{n}+2MU^{n-1}-MU^{n-2}$. Hence, we have reduced the problem of (A146) into solving the linear system (A151), with the solution described above. Furthermore, the matrices *M* (known as the mass matrix) and *K* (known as the stiffness matrix) can be pre-calculated for efficiency.

## Appendix F

This appendix provides some essential definitions used for the characterization of dynamical system models for analysis.

**Definition** **A1** (Dynamical system). *A real-time dynamical system is a tuple (T,M,Φ), where T is a monoid (an algebraic construct, such as an open interval in $R_{+}$). M is a manifold locally diffeomorphic to a Banach space, usually called the *phase space*. As opposed to the configuration space describing the “position” of a dynamical system, the phase space describes the “states” or “motion” of the dynamical system. It is often defined as the tangent bundle TM or the cotangent bundle $T^{*}M$ of the underlying manifold. Φ:T×M⊇U→M, where ${\mathrm{proj}}_{2}\left(U\right)=M$, is the (continuous) evolution function [53].*

A phonation model outputs a phase space trajectory of state variables that describes the movements of the vocal folds. The trajectories tend to fall into orbits with regular or irregular behaviors that explain observed behavior patterns of the vocal folds. The possible types and distributions of these orbits depend on the system parameters.

### Appendix F.1. The Evolution of a Dynamical System

A dynamical system can be instantiated with ordinary or partial differential equations with initial conditions, and the evolution function Φ is the solution to the ODE or PDE.

**Definition** **A2** (Evolution function). *Denote the duration of evolution of a dynamical system as I(x)={t∈T∣(t,x)∈U}. The evolution function Φ is a group action of T on M satisfying*

1. *Φ(0,x)=x,forallx∈M;*
2. *$\Phi \left(t_{2}+t_{1},x\right)=\Phi \left(t_{2},\Phi \left(t_{1},x\right)\right),\;for\;t_{1},t_{2}+t_{1}\in I\left(x\right),t_{2}\in I\left(\Phi \left(t_{1},x\right)\right)$.*

### Appendix F.2. Trajectory, Flow, Orbit and Invariance

Write $\Phi_{x}\left(t\right)\equiv \Phi^{t}\left(x\right)\equiv \Phi \left(t,x\right)$. The map $\Phi^{t}:M\to M$ is a diffeomorphism (i.e., differentiable, invertible, bijection map between manifolds).

**Definition** **A3** (Flow, orbit, invariance). *The map $\Phi_{x}:I\left(x\right)\to M$ is the *flow *or* trajectory* through x. The set of all flows $\gamma_{x}\left\{\Phi_{x}|t\in I\left(x\right)\right\}$ is the *orbit* through x. Particularly, a subset S⊆M is called*Φ-invariant* if Φ(t,x)∈S for all x∈S and t∈T.*

### Appendix F.3. Phase Space Behavior: Attractor

The behaviors of flows can be described by their attractor/attraction sets.

**Definition** **A4** (Attractor). *An attractor set A⊆M in the phase space is a closed subset satisfying for some initial point x, there exists a $t_{0}$ such that $\Phi_{x}\left(t\right)\in A$ for any $t>t_{0}$.*

Namely, the orbit $\gamma_{x}$ is “trapped” in the interior of *A*. A dynamical system can have more than one attractor set depending on the choice of initial points (or the choice of parameters, as we will see later). Locally we can talk about a *basin of attraction*B(A), which is a neighborhood of *A* satisfying for any initial point x∈B(A), and its orbit is eventually trapped in *A*.

There are different types of attractor sets. Some are shown in Figure A1. The simplest one is a *fixed point* or an *equilibrium point*, to which a trajectory in phase space converges regardless of initial settings of the variables (or their starting point). To study vocal fold behaviors, we are particularly interested in those attractors revealing the periodic motion of the flow in phase space. Such attractors include the *limit cycle* or the *limit torus*, which are isolated periodic or toroidal orbits, respectively. Some attractor sets have a fractal structure resultant from a chaotic state of the dynamical system [20,54], and are called *strange attractors*.

### Appendix F.4. Chaos and Exponential Divergence of Phase Space Trajectories

Chaos is a characteristic state of a nonlinear dynamical system. There are different definitions of chaos. A simple one is as follows:

**Definition** **A5** (Chaos). *Equip a distance metric d on the phase space M. Then C∈M is referred to as a chaotic set of Φ if, for any x,y∈C, x≠y, we have*

(A152) $$\begin{array}{cc} \; & \lim_{n\to \infty }\inf d\left(\Phi^{n}\left(x\right),\Phi^{n}\left(y\right)\right)=0 \end{array}$$

(A153) $$\begin{array}{cc} \; & \lim_{n\to \infty }\sup d\left(\Phi^{n}\left(x\right),\Phi^{n}\left(y\right)\right)>0 \end{array}$$

Thus, by definition, chaos is a state characterized by extreme sensitivity to initial conditions (trajectories starting from any two arbitrarily close initial conditions diverge exponentially). The Lypunov exponent is used to quantify this divergence. It also measures the measures the sensitivity of the evolution of the dynamical system to initial conditions.

### Appendix F.5. Stability

Attractor sets (of all types) also characterize the stability of dynamical systems.

**Definition** **A6** (Stability). *A compact* Φ-*invariant subset A=Φ(A)⊆M is called a *Lyapunov stable* attraction set if*

1. *It has an open basin of attraction B(A);*
2. *The Lyapunov stability condition is satisfied: every neighborhood U of A contains a smaller neighborhood V such that every iterative forward image $\Phi^{n}\left(V\right)$ is contained in U.*

### Appendix F.6. Poincaré Map and Poincaré Section

To study the trajectory (or orbit) structure of dynamical systems, we use the Poincaré map or Poincaré section.

**Definition** **A7** (Poincaré map [53]). *For an n-dimensional phase space with a periodic orbit $\gamma_{x}$, a Poincaré section S is an (n−1)-dimensional section (hyper-plane) that is transverse to $\gamma_{x}$. Given an open, connected neighborhood U⊆S of x, the Poincaré map on Poincaré section S is a map P:U→S, $x\mapsto \Phi_{x}\left(t_{s}\right)$ where $t_{s}$ is the time between the two intersections, satisfying*

1. *P(U) is a neighborhood of x and P:U→P(U) is a diffeomorphism;*
2. *For every point x in U, the positive semi-orbit of x intersects S for the first time at P(x).*

### Appendix F.7. Bifurcation

Since the flow of a dynamical system in its phase space is a function of its parameters, the topological structure of the trajectories (including attractor sets) in phase space changes as the parameters change. To see how the topological structure changes with system parameters, we study the bifurcation map of the system.

**Definition** **A8** (Bifurcation). *A bifurcation occurs when a small smooth change in a system parameter value causes an abrupt change in the topological structure of the trajectory in phase space. A *bifurcation diagram* is a visualization of the system’s parameter space showing the number and behavior of attractor sets for each parameter configuration.*

At a *bifurcation point*, the system stability may change as the topological structure splits or merges, such as the periodic doubling or halving of a limit cycle.

## References

[1] Cveticanin L. (2012). Review on Mathematical and Mechanical Models of the Vocal Cord. J. Appl. Math..

[2] Titze I.R. (1988). The physics of small-amplitude oscillation of the vocal folds. J. Acoust. Soc. Am..

[3] Döllinger M., Gómez P., Patel R.R., Alexiou C., Bohr C., Schützenberger A. (2017). Biomechanical simulation of vocal fold dynamics in adults based on laryngeal high-speed videoendoscopy. PLoS ONE.

[4] Herbst C.T., Fitch W., Švec J.G. (2010). Electroglottographic wavegrams: A technique for visualizing vocal fold dynamics noninvasively. J. Acoust. Soc. Am..

[5] Mergell P., Herzel H., Titze I.R. (2000). Irregular vocal-fold vibration—High-speed observation and modeling. J. Acoust. Soc. Am..

[6] Zhang Z. (2016). Mechanics of human voice production and control. J. Acoust. Soc. Am..

[7] Tao C., Zhang Y., Hottinger D.G., Jiang J.J. (2007). Asymmetric airflow and vibration induced by the Coanda effect in a symmetric model of the vocal folds. J. Acoust. Soc. Am..

[8] Erath B.D., Plesniak M.W. (2006). The occurrence of the Coanda effect in pulsatile flow through static models of the human vocal folds. J. Acoust. Soc. Am..

[9] Singh R. (2019). Profiling Humans from Their Voice.

[10] Flanagan J., Landgraf L. (1968). Self-oscillating source for vocal-tract synthesizers. IEEE Trans. Audio Electroacoust..

[11] Ishizaka K., Flanagan J.L. (1972). Synthesis of voiced sounds from a two-mass model of the vocal cords. Bell Syst. Tech. J..

[12] Zhang Z., Neubauer J., Berry D.A. (2006). The influence of subglottal acoustics on laboratory models of phonation. J. Acoust. Soc. Am..

[13] Zhao W., Zhang C., Frankel S.H., Mongeau L. (2002). Computational aeroacoustics of phonation, Part I: Computational methods and sound generation mechanisms. J. Acoust. Soc. Am..

[14] Zhang C., Zhao W., Frankel S.H., Mongeau L. (2002). Computational aeroacoustics of phonation, Part II: Effects of flow parameters and ventricular folds. J. Acoust. Soc. Am..

[15] Lucero J.C. (1993). Dynamics of the two-mass model of the vocal folds: Equilibria, bifurcations, and oscillation region. J. Acoust. Soc. Am..

[16] Lucero J.C., Schoentgen J. (2013). Modeling vocal fold asymmetries with coupled van der Pol oscillators. Proc. Mtgs. Acoust.

[17] Alipour F., Berry D.A., Titze I.R. (2000). A finite-element model of vocal-fold vibration. J. Acoust. Soc. Am..

[18] Yang A., Stingl M., Berry D.A., Lohscheller J., Voigt D., Eysholdt U., Döllinger M. (2011). Computation of physiological human vocal fold parameters by mathematical optimization of a biomechanical model. J. Acoust. Soc. Am..

[19] Pickup B.A., Thomson S.L. (2009). Influence of asymmetric stiffness on the structural and aerodynamic response of synthetic vocal fold models. J. Biomech..

[20] Jiang J.J., Zhang Y., Stern J. (2001). Modeling of chaotic vibrations in symmetric vocal folds. J. Acoust. Soc. Am..

[21] Titze I.R. (2008). Nonlinear source—Filter coupling in phonation: Theory. J. Acoust. Soc. Am..

[22] Story B.H., Titze I.R. (1995). Voice simulation with a body-cover model of the vocal folds. J. Acoust. Soc. Am..

[23] Chan R.W., Titze I.R. (2006). Dependence of phonation threshold pressure on vocal tract acoustics and vocal fold tissue mechanics. J. Acoust. Soc. Am..

[24] Lucero J.C., Schoentgen J., Haas J., Luizard P., Pelorson X. (2015). Self-entrainment of the right and left vocal fold oscillators. J. Acoust. Soc. Am..

[25] Maeda S. (1990). Compensatory articulation during speech: Evidence from the analysis and synthesis of vocal-tract shapes using an articulatory model. Speech Production and Speech Modelling.

[26] Birkholz P., Kröger B.J. Simulation of vocal tract growth for articulatory speech synthesis. Proceedings of the 16th International Congress of Phonetic Sciences.

[27] Dang J., Honda K. (2004). Construction and control of a physiological articulatory model. J. Acoust. Soc. Am..

[28] Portnoff M.R. (1973). A Quasi-One-Dimensional Digital Simulation for the Time-Varying Vocal Tract. Ph.D. Thesis.

[29] Allen D.R., Strong W.J. (1985). A model for the synthesis of natural sounding vowels. J. Acoust. Soc. Am..

[30] Motoki K., Pelorson X., Badin P., Matsuzaki H. Computation of 3-D vocal tract acoustics based on mode-matching technique. Proceedings of the Sixth International Conference on Spoken Language Processing.

[31] Zhao W., Singh R. Speech-based parameter estimation of an asymmetric vocal fold oscillation model and its application in discriminating vocal fold pathologies. Proceedings of the ICASSP 2020-2020 IEEE International Conference on Acoustics, Speech and Signal Processing (ICASSP).

[32] Erath B.D., Plesniak M.W. (2006). An investigation of jet trajectory in flow through scaled vocal fold models with asymmetric glottal passages. Exp. Fluids.

[33] Eisner E. (1967). Complete solutions of the “Webster” horn equation. J. Acoust. Soc. Am..

[34] Titze I.R., Martin D.W. (1998). Principles of voice production. Acoust. Soc. Am. J..

[35] Alku P. (2011). Glottal inverse filtering analysis of human voice production—A review of estimation and parameterization methods of the glottal excitation and their applications. Sadhana.

[36] Morse P.M., Ingard K.U. (1986). Theoretical Acoustics.

[37] Steinecke I., Herzel H. (1995). Bifurcations in an asymmetric vocal-fold model. J. Acoust. Soc. Am..

[38] Bhat C., Kopparapu S.K. FEMH Voice Data Challenge: Voice disorder Detection and Classification using Acoustic Descriptors. Proceedings of the 2018 IEEE International Conference on Big Data (Big Data).

[39] Al Ismail M., Deshmukh S., Singh R. Detection of COVID-19 through the analysis of vocal fold oscillations. Proceedings of the ICASSP 2021-2021 IEEE International Conference on Acoustics, Speech and Signal Processing (ICASSP).

[40] Deshmukh S., Al Ismail M., Singh R. Interpreting glottal flow dynamics for detecting COVID-19 from voice. Proceedings of the ICASSP 2021-2021 IEEE International Conference on Acoustics, Speech and Signal Processing (ICASSP).

[41] Zhang J. (2022). Vocal Fold Dynamics for Automatic Detection of Amyotrophic Lateral Sclerosis from Voice. Master’s Thesis.

[42] Lee K.B., Kim J.H. Mass-spring-damper motion dynamics-based particle swarm optimization. Proceedings of the 2008 IEEE Congress on Evolutionary Computation (IEEE World Congress on Computational Intelligence).

[43] Do Carmo M.P., Flaherty Francis J. (1992). Riemannian Geometry.

[44] Kantorovich L.V., Akilov G.P. (2016). Functional Analysis.

[45] Zhu K. (2007). Operator Theory in Function Spaces.

[46] Giles M.B., Süli E. (2002). Adjoint methods for PDEs: A posteriori error analysis and postprocessing by duality. Acta Numer..

[47] Dong C., Jin Y. (2012). MIMO nonlinear ultrasonic tomography by propagation and backpropagation method. IEEE Trans. Image Process..

[48] Langtangen H.P., Mardal K.A. (2019). Introduction to Numerical Methods for Variational Problems.

[49] Ames W.F. (2014). Numerical Methods for Partial Differential Equations.

[50] Thomée V. (1984). Galerkin Finite Element Methods for Parabolic Problems.

[51] Larson M.G., Bengzon F. (2010). The finite element method: Theory, implementation, and practice. Texts Comput. Sci. Eng..

[52] Alnæs M., Blechta J., Hake J., Johansson A., Kehlet B., Logg A., Richardson C., Ring J., Rognes M.E., Wells G.N. (2015). The FEniCS project version 1.5. Arch. Numer. Softw..

[53] Birkhoff G.D. (1927). Dynamical Systems.

[54] Jiang J.J., Zhang Y. (2002). Chaotic vibration induced by turbulent noise in a two-mass model of vocal folds. J. Acoust. Soc. Am..

